# Emerging Theranostic Radiometals (^149^Tb, ^44^Sc, ^52^Mn, ^203^Pb, ^55^Co)—Decay Diversity, Production Landscape, and Translational Imaging

**DOI:** 10.3390/ph19060889

**Published:** 2026-06-03

**Authors:** Noeen Malik, Yashas Ullas Lokesha, Frezghi G. Habte, Heike E. Daldrup-Link

**Affiliations:** 1 Cyclotron and Radiochemistry Facility, Department of Radiology, Stanford University School of Medicine, Palo Alto, CA 94304, USA; noeen254@stanford.edu; 2 Molecular Imaging Program at Stanford (MIPS), Department of Radiology, Stanford University School of Medicine, Stanford, CA 94305, USA; yash1502@stanford.edu (Y.U.L.); fhabte@stanford.edu (F.G.H.)

**Keywords:** PET, SPECT, imaging, diagnosis, oncology, neuroimaging, radioisotopes, terbium-149, scandium-44, manganese-52, lead-203, cobalt-55, radiochemistry, radiometals, theranostic

## Abstract

Emerging metallic radionuclides are expanding theranostic capabilities in nuclear medicine by improving diagnostic sensitivity, enabling dosimetry, and matched theranostic approaches. ^149^Tb, ^44^Sc, ^52^Mn, ^203^Pb, and ^55^Co offer distinct nuclear decay properties, including extended half-lives, variable positron emissions, and prompt γ-photons that may influence quantitative imaging performance. Cyclotron and generator routes integrating enriched targets and optimized separations support clinical-scale supply, while advances in chelation chemistry improve in vivo stability and imaging performance. Preclinical and early clinical data demonstrate that ^149^Tb provides intrinsic α-therapy and PET imaging capability for theranostic use, ^44^Sc enables extended imaging relative to ^68^Ga, supporting delayed imaging and improved tumor-to-background contrast for peptide-based radiopharmaceuticals and theranostic applications. ^52^Mn supports prolonged biological tracking for antibody- and engineered-protein-targeted studies, whereas ^203^Pb serves as a diagnostic surrogate for ^212^Pb based α-therapy (*via* ^212^Bi). ^55^Co PET imaging supports the development and evaluation of ^58m^Co Auger electron therapy. Current challenges include limited global availability of highly enriched targets, management of long-lived radioactive by-products, and the need for standardized dosimetry and regulatory pathways to ensure reproducibility and safety. Ongoing developments in automated target handling, optimized separations, next-generation chelators, and harmonized regulation may facilitate broader clinical translation.

## 1. Introduction

The practice of nuclear medicine has undergone a fundamental evolution from primarily diagnostic imaging toward integrated theranostic paradigms, in which radionuclides are used not only to visualize disease but also to guide and deliver targeted therapy. This transformation has been driven by the clinical success of receptor-targeted radiopharmaceuticals, such as ^68^Ga-labeled peptides for imaging and ^177^Lu-based agents for therapy, which have demonstrated the value of coupling molecular targeting with patient-specific dosimetry. However, limitations in radionuclide availability, production scalability, and physicochemical compatibility with biological vectors, imaging time windows, and coordination chemistry continue to restrict optimal matching between diagnostic and therapeutic radionuclides, particularly for biological vectors with slower pharmacokinetics such as monoclonal antibodies [1].

**Figure 1:**
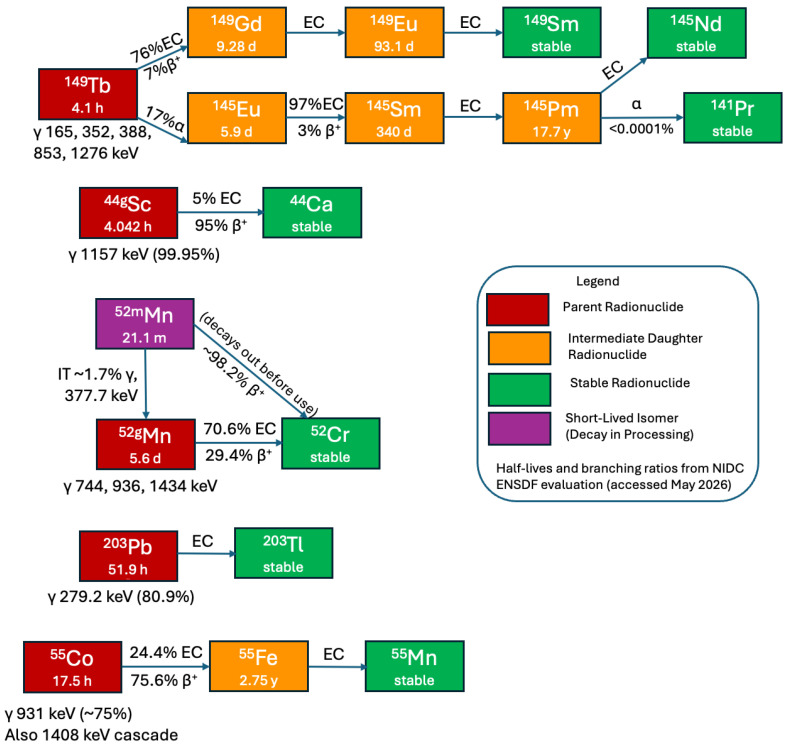
Decay schemes of radionuclides reviewed such as ^149^Tb, ^44^Sc, ^52^Mn, ^203^Pb, and ^55^Co.

To address these limitations, increasing attention has focused on emerging radiometals that offer improved alignment between radionuclide decay properties (Figure 1) and biological behavior. These radionuclides expand the theranostic toolkit by providing a broader range of half-lives, emission profiles (β^+^, α, Auger electrons, and γ), and coordination chemistries that enable stable conjugation to diverse targeting vectors. In parallel, international initiatives such as PRISMAP and CERN-MEDICIS are advancing to improved access, production capacity and distribution capabilities for non-conventional radionuclides, addressing long-standing limitations in availability and scalability [2].

Theranostic nuclear medicine increasingly draws on a wide chemical and physical palette, spanning non-metal positron emitters (e.g., ^18^F, ^11^C, ^13^N, ^15^O), halogen β^+^/α emitters (e.g., ^124^/^131^I, ^76/77^Br, ^211^At), and a growing family of d- and f-block radiometals. Non-metals are attractive for small-molecule labeling via covalent C–X bonds, whereas radiometals enable chelator-based attachment to peptides, antibodies, and nanoparticles, with orthogonal control over pharmacokinetics through ligand design. Within the radiometal subset, theranostic deployment requires either a single isotope that combines diagnostic and therapeutic emissions, or a matched diagnostic/therapeutic pair sharing identical coordination chemistry. The five radiometals addressed here, ^149^Tb, ^44^Sc, ^52^Mn, ^203^Pb and ^55^Co, span both modalities and are highlighted because each illustrates a distinct decay-physics niche that is now reaching translational maturity.

A wide spectrum of radionuclides, including ^61^Cu [3] and ^43^Sc [4], has been proposed as next-generation PET imaging agents. While these candidates exhibit favorable nuclear characteristics, their broader clinical impact remains constrained by challenges related to production scalability, coordination chemistry, or limited translational validation. In contrast, the radiometals selected in this review—^149^Tb, ^44^Sc, ^52^Mn, ^203^Pb, and ^55^Co—represent a focused group distinguished by three complementary attributes: (i) relevance to theranostic strategies (either intrinsic or paired); (ii) half-life compatibility with biologically relevant targeting vectors (small molecules, peptides, and antibodies); and (iii) demonstrated progression along the translational spectrum from preclinical studies to early clinical evaluation.

Importantly, these radionuclides span a continuum of theranostic functionality rather than representing a uniform class. At one end of this spectrum, ^149^Tb uniquely integrates diagnostic and therapeutic capabilities within a single isotope, emitting both α-particles for targeted alpha therapy and β^+^ particles for PET imaging (“α-PET”). In contrast, radionuclides such as ^44^Sc and ^203^Pb function as diagnostic partners to established therapeutic counterparts (^47^Sc and ^212^Pb, respectively), enabling matched-pair theranostic strategies that support dosimetry and treatment planning.

While both (^44g^Sc and ^44m^Sc) have been investigated, current clinical and translational efforts primarily focus on ^44g^Sc due to its favorable positron emission characteristics and compatibility with routine cyclotron production, whereas ^44m^Sc remains of interest for extended imaging or generator-based concepts but has not yet achieved comparable translational maturity.

^55^Co represents an emerging component of cobalt-based theranostic systems, particularly in conjunction with ^58m^Co for Auger electron therapy, although this paradigm remains under development. Finally, ^52^Mn primarily contributes through its extended half-life, which enables imaging of slow biological processes such as antibody distribution and cellular trafficking, thereby supporting longitudinal evaluation of therapeutic agents rather than serving as a direct theranostic pair.

Within the terbium family, multiple isotopes, including ^149^Tb, ^152^Tb, ^161^Tb, and ^155^Tb, have been investigated for diagnostic and theranostic applications. Accordingly, ^149^Tb was selected over other terbium isotopes because it uniquely combines α-emission and positron emission within a single radionuclide, enabling simultaneous therapy and PET imaging. In contrast, ^152^Tb is a pure positron emitter suited for diagnostic PET imaging. ^161^Tb (t_½_ ≈ 6.89 d) is a β^−^ and Auger-electron emitter pursued as a therapeutic counterpart to ^177^Lu, with first-in-human data in mCRPC (VIOLET trial) while ^155^Tb is a γ-emitter used for SPECT imaging. Although these isotopes are integral components of the broader terbium theranostic “quadruplet,” they function predominantly as diagnostic companions rather than standalone theranostic agents.

Consistent with this framework, this review emphasizes radionuclides that either intrinsically integrate therapeutic and imaging capabilities or support therapeutic planning and evaluation through matched radionuclide pairs or biologically relevant imaging windows.

Beyond their theranostic relevance, the selected radionuclides also represent a diverse spectrum of coordination chemistry and nuclear decay behavior, encompassing hard trivalent metals (lanthanide (^149^Tb), rare-earth metal (^44^Sc)), transition metals (^52^Mn, ^55^Co), and post-transition metals (^203^Pb). This diversity enables systematic evaluation of key parameters influencing radiopharmaceutical performance, including chelator selection, radiolabeling kinetics, in vivo stability, and imaging characteristics.

Despite their promise, several challenges must be addressed to enable broader clinical translation. Production of many of these radionuclides relies on enriched target materials and solid-target cyclotron irradiation, which introduce constraints related to cost, infrastructure, and recycling efficiency. Radionuclidic purity remains a critical consideration, as long-lived impurities can impact both radiation dose and regulatory acceptance. From a radiochemical perspective, the diverse electronic structures of these metals necessitate metal-specific chelator development to ensure kinetic inertness and in vivo stability, as conventional systems such as DOTA are not universally optimal across all radiometals. Furthermore, from an imaging standpoint, several of these radionuclides emit high-energy prompt γ-photons in addition to positrons, increasing random coincidences and dead-time effects and thereby complicating quantitative PET imaging unless appropriate correction models are implemented.

**Figure 2:**
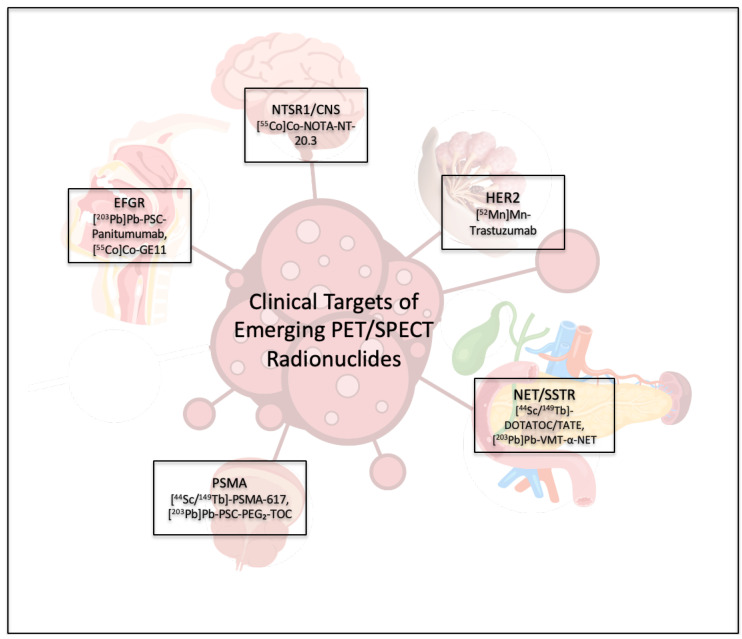
Clinical significance and targets of emerging diagnostic radionuclides.

Taken together, these considerations highlight the need for a comprehensive and balanced evaluation of emerging radiometals that integrates production feasibility, radiochemical performance, and translational readiness. This review presents a structured comparison of ^149^Tb, ^44^Sc, ^52^Mn, ^203^Pb, and ^55^Co, focusing on production strategies, radiolabeling chemistry, and translational applications. By positioning these radionuclides within the broader context of established imaging agents such as ^68^Ga and ^64^Cu, we aim to clarify their distinct roles, current limitations, and realistic pathways toward clinical integration. The complementary clinical landscape, the principal disease indications and molecular targets, namely somatostatin receptor type 2 (SSTR2), prostate-specific membrane antigen (PSMA), neurotensin receptor type 1 (NTSR-1), and human epidermal growth factor receptor 2 (HER2), for which each of these radiometals is most relevant, is summarized in Figure 2, which together with Figure 1 motivates the radiometal-by-radiometal review that follows.

A property shared by four of the five radiometals reviewed here, ^149^Tb, ^44^Sc, ^52^Mn and ^55^Co, is the emission of high-energy prompt γ photons with branching ratios that are non-trivial relative to the diagnostic 511 keV annihilation line. This has three practical consequences: (i) facility shielding optimized for ^99m^Tc, ^18^F or ^64^Cu is generally insufficient and must be upgraded; (ii) Compton scatter and detector dead-time degrade quantification at standard injected activities; and (iii) staff dose during preparation and administration is elevated compared with conventional PET nuclides. Long-axial-field-of-view (LAFOV/total-body) PET scanners, which offer approximately an order-of-magnitude increase in geometric sensitivity, are emerging as a key enabling technology for safely deploying these radiometals at low injected activity. In parallel, the same prompt-γ component is now being exploited positively in positronium-lifetime imaging, recently demonstrated for ^52^Mn and ^55^Co, opening a new functional-imaging modality. ^203^Pb is the exception, with a single 279 keV γ emission well-suited to existing SPECT instrumentation.

## 2. Terbium-149

Terbium-149 (^149^Tb) is a dual-emitting radiotracer with 4.12 h half-life that enables targeted alpha therapy (α-emission = 16.7%) (TAT) with simultaneous PET imaging through co-emitted β^+^-particles (7.1% emission) [5], supporting dosimetry and therapy monitoring. Terbium belongs to the “Swiss Army knife” lanthanide family with isotopes for α, β^−^, Auger, and γ theranostics [6]. The decay scheme of ^149^Tb (β^+^ 7.1%, α 17%, EC 76.2%; principal γ 165, 352, 388, 853, and 1276 keV) is summarized in Figure 1. The relatively low α-branching ratio, compared with the near-quantitative α-emission of ^225^Ac or ^211^At, dilutes the therapeutic α dose per decay and contributes to the off-target dose via the accompanying β^+^/EC/γ channels, and is a key practical consideration for any ^149^Tb-based α-therapy regimen.

### 2.1. Production

^149^Tb has, to date, been produced primarily through proton-induced spallation of tantalum targets with subsequent on-line isotope separation (ISOL) at high-energy (GeV-class; High energy > 100 MeV) facilities, most notably ISOLDE/CERN operating within the CERN-MEDICIS framework; the post-separation radiochemical purification and formulation as [^149^Tb]TbCl_3_ in 0.05 M HCl is routinely performed at the Paul Scherrer Institute (PSI) [5]. Anticipated commissioning of the planned IMPACT/TATTOOS program at PSI is intended to provide additional on-site spallation production capacity over the coming years. These CERN-MEDICIS/PSI collaborations have supplied much of research-grade ^149^Tb used in preclinical theranostic studies, with batch activities typically of the order of tens to ~100 MBq per run [5,7]. Among lower-energy alternatives, the ^152^Gd(p,4n)^149^Tb and ^151^Eu(^3^He,5n)^149^Tb reactions at ~35–60 MeV fall within the proton and ^3^He beam capabilities of multi-particle, intermediate-energy cyclotrons such as ARRONAX (Nantes, France), positioning these facilities as candidate sites for expanding cyclotron-based ^149^Tb production [5,7]. Consequently, routine use of ^149^Tb is effectively restricted to a small number of expert centers in Europe, and broader clinical deployment will require either replication of ISOL infrastructure or significant improvements in cyclotron-based production yields [5,7]. Complementary cross-section measurements of ^147–149^Sm(^6^Li,x) provide useful nuclear data, although preferential population of ^149m^Tb, which does not decay to the therapeutically relevant ground state, limits their practical utility [8].

From a dosimetry perspective, ^149^Tb differs from actinium-based α-emitters in that it does not generate long-lived α-emitting daughters. However, long-lived non-α progeny remain relevant for dosimetry and potential redistribution [5]. However, ^149^Tb decays by α-emission in only ~16.7% of disintegrations (to ^145^Eu) and by electron capture/β^+^ in the majority of events (to ^149^Gd); because α-recoil can displace the daughter from the chelator, redistribution of intermediate-to-long-lived progeny (including ^145^Eu, ^149^Gd and subsequent nuclides with half-lives ranging from days to years) represents a real dosimetry consideration that should be addressed in translational planning [5,7]; comparable considerations have been detailed for the safety profile of ^225^Ac-based α-therapy [9]. The short physical half-life (t_½_ = 4.12 h) and the absence of a suitable long-lived parent radionuclide also limit the feasibility of generator-based production systems [5].

As part of broader lanthanide-separation strategies relevant to terbium purification, P204–P507 solvent-impregnated resins supported on Amberlite XAD-7HP have demonstrated efficient separation of Gd(III), Tb(III), and Dy(III) in nitric acid systems [10]. Optimal adsorption of Tb (III) was observed at 0.01 mol/L HNO_3_, with adsorption capacities reaching ~156 μg/g at a solid–liquid ratio of 100 mg/mL and contact time of 180 min [10]. Efficient desorption of Tb(III) was achieved using 1 mol/L nitric acid [10]. Taken together, the principal practical limitations of ^149^Tb production at the time of writing are (i) restricted access to GeV-class proton accelerators and ISOL mass-separation infrastructure, (ii) modest batch activities relative to α-therapy requirements, (iii) short physical half-life constraining distribution networks, and (iv) dosimetry uncertainty associated with α-recoil-induced redistribution of daughter nuclides [5,7,10]. These limitations and mitigation strategies are consistent with the multicenter recommendations of the EU SECURE Project on α-particle therapy [11].

### 2.2. Radiolabeling

Somatostatin receptors, predominantly SSTR2, are over-expressed on most gastro-entero-pancreatic and bronchial neuroendocrine tumors. DOTA-conjugated somatostatin analogues such as DOTATOC, DOTATATE and DOTA-LM_3_ are therefore the established peptide vectors for both diagnostic imaging (e.g., [^68^Ga]Ga-DOTATATE) and peptide-receptor radionuclide therapy (e.g., [^177^Lu]Lu-DOTATATE), and these are the conventional chelators for translational evaluation of new lanthanide radiometals such as ^149^Tb. Chelator selection for ^149^Tb is governed by the hard, trivalent, and lanthanide-like coordination behavior of Tb(III), which favors octa-dentate oxygen/nitrogen donor macrocycles [12]. DOTA is the most widely applied chelator for ^149^Tb, and the stable complexation of this radiolanthanide with DOTA has been highlighted as a favorable feature for theranostic applications [12]. Radiolabeling of somatostatin analogues with [^149^Tb]TbCl_3_ was performed under standard labeling conditions at pH 4.5 (ammonium acetate buffer), typically at 95 °C for 10–15 min, yielding molar activities of up to 20 MBq/nmol with radiochemical purity ≥ 98% [5]. Comparable labeling conditions have been reported for [^149^Tb]Tb-PSMA-617, while DOTA-based somatostatin analogues ([^149^Tb]Tb-DOTA-LM_3_) have been evaluated under similar conditions, supporting DOTA as the current benchmark chelator for ^149^Tb [5,13]. Beyond DOTA, however, the coordination chemistry of Tb^3+^ is shared by a broader family of lanthanide-compatible ligands that have been validated with Tb congeners (Tb^3+^, Gd^3+^, Lu^3+^) and are chemically applicable to ^149^Tb. These include expanded-cavity macrocycles such as MACROPA, pyridyl–picolinate scaffolds (e.g., H_4_pypa), phosphonate-based aza-macrocycles, and 6-amino-6-methyl-perhydro-1,4-diazepine-*N*,*N*,*N′*,*N′*-tetraacetic acid (AAZTA)-derived ligands, each of which has been shown to accommodate trivalent lanthanide cations with high kinetic inertness under mild labeling conditions. Direct ^149^Tb labeling data with these newer scaffolds remain limited, but their performance with Tb^3+^/Sc^3+^/Lu^3+^ supports their consideration as credible alternatives for next-generation ^149^Tb radiopharmaceuticals when mild, fast, or room-temperature labeling is required [12].

Recent reviews further emphasize the importance of rapid complexation and fast targeting strategies to mitigate challenges associated with short-lived radionuclides and α-emitter recoil, highlighting the potential role of click chemistry and pre-targeting approaches in this context [14]. In particular, bioorthogonal inverse electron-demand Diels–Alder (IEDDA) ligation between tetrazines and trans-cyclooctenes (TCO) has emerged as a powerful strategy for rapid, mild, and site-specific bioconjugation under physiological conditions [15,16]. The TCO–tetrazine reaction exhibits exceptionally fast kinetics and has been widely applied in radiochemistry and pre-targeted imaging and therapy using trivalent radiometals [15,16]. While applications specifically employing ^149^Tb in tetrazine–TCO click labeling remain limited, the demonstrated compatibility of DOTA-based clickable chelators with trivalent radiometals supports the potential application of this methodology to terbium [15,16].

In this approach, a tumor-targeting biomolecule is pre-functionalized with either a tetrazine or a trans-cyclooctene (TCO), administered to the subject, allowed to localize on the target, and is then captured in vivo by the complementary partner carrying the radiometal–chelator complex via an inverse electron-demand Diels–Alder (IEDDA) ligation. The reaction is bio-orthogonal, proceeds at room temperature in aqueous media with second-order rate constants up to 10^6^ M^−1^ s^−1^, and is particularly attractive for short-lived radiometals such as ^149^Tb because the radiolabeled, low-molecular-weight tetrazine clears rapidly and is captured selectively on the pre-localized vector.

Based on the available evidence, DOTA remains the most extensively validated chelator for ^149^Tb and is recommended for near-term clinical translation of somatostatin- and PSMA-targeted tracers [5,12,13]. Other chemically plausible scaffolds, including MACROPA, H_4_pypa, and AAZTA-derivatives, represent promising alternatives, but require isotope-specific validation for ^149^Tb-compatibility, particularly for labeling heat-sensitive vectors (e.g., antibodies, antibody fragments) [12]. These chelator considerations align with the broader terbium radionuclide family, for which the translational progress of the ^161^Tb β^−^/Auger pair has been recently reviewed [17].

### 2.3. Translational Applications

The radioisotope ^149^Tb has been investigated preclinically as a potential alternative to currently employed α-emitters for TAT [12] because of its low-energy α-particle emission (3.97 MeV, α-emission = 16.7%), corresponding to a tissue range of ~25 μm and a high linear energy transfer (LET = 140 keV/μm) [12]. In addition, ^149^Tb provides positron emissions (E_β+mean_ = 730 keV, I_β+_ = 7.1%) for PET imaging and γ-rays (E_γ_ = 165 keV, 26.4%) for SPECT imaging. Several authors described the combination of ^149^Tb-TAT with either PET or SPECT imaging [5,12,13,18]. For example, in vitro and in vivo studies demonstrated that ^149^Tb-labeled somatostatin receptor (SSTR)-targeting agents (agonist DOTATATE and antagonist DOTA-LM_3_) reduced tumor cell viability without evidence of significant acute toxicity in preclinical models [5]. Similarly, [^149^Tb]PSMA-617 significantly delayed tumor growth in a mouse model of prostate-specific membrane antigen (PSMA)-expressing prostate cancer [13], significantly extending survival in treated mice (36 days) compared to untreated controls (20 days). In a severe combined immunodeficient (SCID) leukemia model, [^149^Tb]rituximab (anti-CD20 antibody, 1.11 GBq/mg) administered two days after intravenous injection of 5 × 10^6^ Daudi cells achieved 89% tumor-free survival beyond 120 days, while all controls developed lymphoma [18]. A relatively high daughter radioactivity concentration was observed in the spleen of treated animals, likely reflecting reticuloendothelial system involvement and antibody-associated biodistribution patterns, and is consistent with α-recoil-induced redistribution of longer-lived progeny from the originally administered radioconjugate [18].

In vitro, ^149^Tb-labeled SSTR-targeting peptides retained high receptor-binding affinity and demonstrated dose-dependent reduction in tumor cell viability with an α-particle-specific cytotoxicity profile comparable to values reported for other α-emitting radioconjugates, including ^225^Ac and ^213^Bi analogues of the same vectors [5,12]. Similar in vitro reductions in clonogenic survival were observed for [^149^Tb]PSMA-617 in PSMA-positive prostate cancer cell lines [13], indicating that ^149^Tb can deliver therapeutic α-doses indicating effective α-particle-mediated cytotoxicity consistent with established α-emitters while simultaneously enabling PET imaging.

**Figure 3:**
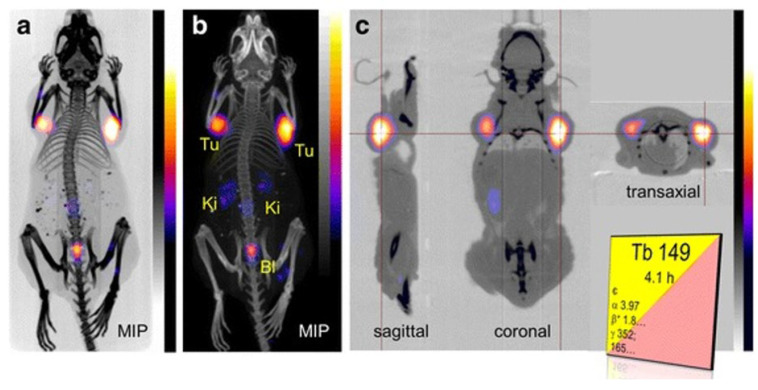
[^149^Tb]DOTANOC PET/CT (7 MBq) in an AR42J mouse at 2 h shows tumor uptake (Tu) with residual radioactivity in kidney (Ki) and bladder (Bl) ((**a**,**b**)—MIP (Maximal intensity projections); (**c**)—sections); decay scheme per Karlsruhe Nuclide Chart [12].

Beyond therapy, ^149^Tb served as a PET imaging tracer via β^+^-emission. PET/CT imaging demonstrated selective accumulation of [^149^Tb]PSMA-617 in PC-3 PIP tumor xenografts, enabling real-time tracer biodistribution and tumor monitoring [13]. PET imaging with [^149^Tb]DOTANOC demonstrated tumor uptake in AR42J (pancreatic cancer) mouse models (Figure 3), with rapid renal excretion of the radiotracer [12]. Compared with established reference agents such as [^68^Ga]Ga-DOTATATE and [^177^Lu]Lu-PSMA-617, [^149^Tb]Tb-DOTATATE and [^149^Tb]Tb-PSMA-617 demonstrate favorable tumor-to-background contrast on early PET imaging, consistent with established radioligand distributions, while simultaneously providing therapeutic α-particle doses, supporting the distinctive imaging-plus-therapy role of ^149^Tb rather than a pure diagnostic replacement [5,12,13]. First-in-human clinical data for the related ^161^Tb β^−^/Auger-emitter in metastatic castration-resistant prostate cancer (mCRPC; VIOLET trial) further underline the clinical maturity of terbium-based radioligand therapy [19]. Although dedicated phantom studies for ^149^Tb remain scarce relative to ^203^Pb and ^44^Sc, the low β^+^ branching (7.1%) has been consistently identified in preclinical imaging work as the principal sensitivity-limiting factor, a finding that should guide future phantom-based harmonization efforts [5,12]. Further studies are needed to optimize ^149^Tb production and to evaluate long-term off-target toxicities associated with α-recoil-induced daughter detachment and redistribution. Pharmacokinetic and dosimetric benchmarking with the ^161^Tb-SibuDAB first-in-human study (PROGNOSTICS phase Ia) provides a useful translational reference framework for terbium-based radiopharmaceutical development [20].

## 3. Scandium-44

Scandium-44 has progressed to early clinical evaluation as a PET radiometal that extends the imaging window beyond ^68^Ga and supports matched-pair theranostic development with therapeutic radiometals such as ^47^Sc and ^177^Lu. Two nuclear states must be distinguished. The ground state ^44g^Sc (t_½_ = 4.04 h, β^+^ = ~94%, Eγ = 1157 keV) is the imaging-relevant isotope. The long-lived metastable isomer ^44m^Sc (t_½_ = 58.6 h) decays predominantly by internal transition to ^44g^Sc and acts as an in vivo generator, extending the effective imaging window for slowly distributing vectors without serial administration [21,22]. For clarity, all references to “^44^Sc” in this review denote the PET-relevant ground state ^44g^Sc unless explicitly noted. With its 4.04 h half-life, high β^+^ branching, and prompt 1157 keV γ-emission, ^44g^Sc offers extended imaging windows, centralized production logistics, and compatibility with theranostic paradigms [21,22], while control of the ^44m^Sc content remains a key radionuclidic-purity consideration during production. These nuclear characteristics support both cyclotron- and generator-based supply chains. The ^44^Sc decay scheme is provided in Figure 1.

### 3.1. Production

The primary production route via cyclotron remains the ^44^Ca(p,n)^44g^Sc reaction on enriched ^44^CaO or ^44^CaCO_3_ targets, which provides high radionuclidic purity and GBq-scale yields compatible with hospital-based workflows [21,23]. This route is currently operated at several academic medical cyclotrons in Europe and North America, including the Paul Scherrer Institute, the University of Wisconsin, and production facilities affiliated with the PRISMAP consortium, where reported physical yields are of the order of hundreds of MBq to GBq scale under medical cyclotron conditions, with energy selection in the ~9–13 MeV window that is chosen specifically to minimize co-production of the long-lived isomer ^44m^Sc [21,23]. Enriched target costs, availability of ^44^Ca, and target processing capacity remain the principal infrastructural limitations for broader deployment [21,23]. Titanium-based routes, including ^47^Ti(p,α)^44g^Sc, are under investigation, but are at an earlier stage and require further optimization to manage yield and radionuclidic purity [24]. Practical implementation at medical cyclotrons has been validated, supporting routine production [23]. Generator-based production via long-lived ^44^Ti/^44g^Sc systems (t_½_(^44^Ti) = 60 y) provides a decentralized alternative, with ongoing improvements in separation chemistry and column design (SnO_2_- and resin-based formats) enhancing robustness and elution performance; however, commercial ^44^Ti/^44^Sc generators are not yet routinely available for clinical use, and access depends largely on research collaborations with accelerator facilities capable of long-term ^44^Ti production [22,24]. From a chemical processing perspective, recent advances in dry thermal separation of scandium (^47^Sc) from irradiated titanium matrices represent vacuum-driven volatilization strategies, reducing aqueous processing burdens and thus, offer potential pathways to be explored in future in target recovery and recycling efficiency for ^44^Sc production [25].

Effective separation of Sc^3+^ from bulk Ca^2+^ matrices is central to achieving clinically useful molar activities. Standard workflows involve target dissolution in dilute HCl followed by extraction chromatography on branched-*N*,*N*,*N′*,*N′*-tetra-n-octyl-diglycolamide (branched-DGA) or UTEVA (di-pentyl-pentylphosphonate) resins, with Ca^2+^ breakthrough monitored to limit metal carryover that would otherwise compete with Sc^3+^ during subsequent chelator labeling [21,23,24]. For generator-based workflows, Sc/Ti separation exploits the large affinity difference of these cations for hydroxamate and DGA resins, with elution of ^44g^Sc in small volumes of dilute HCl or acetate buffer directly compatible with downstream radiolabeling [22,24]. Routine performance criteria include Ca^2+^ content well below chelator stoichiometric thresholds and minimal Ti breakthrough for generator eluates [22,23,24].

Beyond proton-induced pathways, International Atomic Energy Agency (IAEA) assessments underscore the growing strategic interest in photonuclear routes for medical radionuclides, including scandium isotopes (notably ^47^Sc), though clinical integration remains emergent [26]. Such developments support broader clinical integration.

### 3.2. Radiolabeling

Sc^3+^ is a hard trivalent cation that forms stable complexes with oxygen- and nitrogen-donor chelators. Its small ionic radius and oxophilicity influence complexation kinetics and stability during radiolabeling [27,28]. DOTA remains the clinical translational benchmark chelator for ^44g^Sc and its therapeutic congener ^47^Sc, offering high thermodynamic stability and kinetic inertness under physiological conditions [23]. Representative DOTA-based ^44g^Sc radiolabeling is performed at pH 4–5 (acetate or ammonium acetate buffer) and 90–95 °C for 10–20 min, reproducibly achieving apparent molar activities of 10–40 MBq/nmol, radiochemical purities ≥ 95%, and overall labeling efficiencies typically above 90% after solid-phase cleanup [23]. However, the elevated temperatures typically required for DOTA complexation have motivated the development of alternative chelators optimized for milder labeling conditions. AAZTA-derived ligands (e.g., AAZTA5) enable more rapid complexation at room temperature (pH ~4, <5 min), with favorable radiolabeling efficiency and stability compared to those obtained with DOTA and with favorable in vitro stability, facilitating kit-based formulations and labeling of heat-sensitive biomolecules [29].

Next-generation ligand systems further refine the coordination landscape. Phosphonate-based aza-macrocycles enable efficient room-temperature complexation with high apparent molar activities (>100 MBq/nmol in model systems) [27]. Pyridyl–picolinate scaffolds (H_4_pypa and related H_4_py4pa derivatives) exhibit rapid complex formation across a broad pH window (pH 2–5.5) at ambient temperature with excellent serum stability, including successful translation into PSMA-targeted constructs with radiochemical yields > 90% and apparent molar activities suitable for clinical PET imaging [28].

Methodological innovation has also expanded beyond solution-phase chemistry. Solid-phase radiometalation photorelease (SPRP) has been adapted to ^44g^Sc, allowing resin capture, storage flexibility, and photochemical release with high radiochemical purity, validated in preclinical ^44^Sc-PSMA studies ([^44^Sc]Sc-AAZTA-Glu-PSMA-617 and [^44^Sc]Sc-DOTA-Lys-PSMA-617) [30]. Across these platforms, apparent molar activities in the range of 20–50 MBq/nmol and radiochemical purities ≥ 95% are now routinely achievable for ^44g^Sc, approaching the performance of ^68^Ga analogues [23,28,29,30].

On the basis of clinical translation to date and the breadth of published radiolabeling data, DOTA is recommended as the current default chelator for ^44g^Sc applications that tolerate elevated labeling temperatures (e.g., small peptides), while AAZTA5 and H_4_pypa are the most attractive chelators for antibody- and antibody-fragment-based vectors or for decentralized kit-type formulations [23,28,29,30]. Continued validation of SPRP and phosphonate-based aza-macrocycles is warranted for centralized radiopharmacy workflows [27,30].

### 3.3. Translational Applications

**Figure 4:**
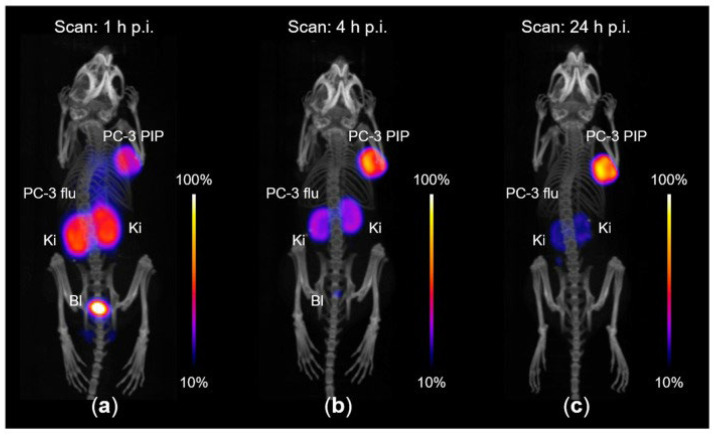
MIP PET/CT of PC-3 PIP/flu tumor-bearing mice after [^44^Sc]Sc-PSMA-ALB-02 (~5 MBq, 1 nmol) i.v.: (**a**) 1 h, (**b**) 4 h, (**c**) 24 h p.i.; scales adjusted for tumor/kidney visibility. PC-3 PIP = PSMA^+^ (right shoulder), PC-3 flu = PSMA^−^ (left), Ki = kidney, Bl = bladder [23] (Licensee MDPI, distributed under the terms and conditions of the Creative Commons Attribution (CC BY) license).

A defining advantage of ^44^Sc over the shorter-lived ^68^Ga is the ability to image at later time points, when tumor accumulation has matured and background activity has cleared. This is illustrated by preclinical PET/CT of PSMA-positive xenografts [23], in which the albumin-binding analogue [^44^Sc]Sc-PSMA-ALB-02 showed improved tumor-to-background contrast at delayed imaging time points up to 24 h post-injection, supporting the suitability of longer half-life of ^44^Sc for delayed imaging (Figure 4).

**Figure 5:**
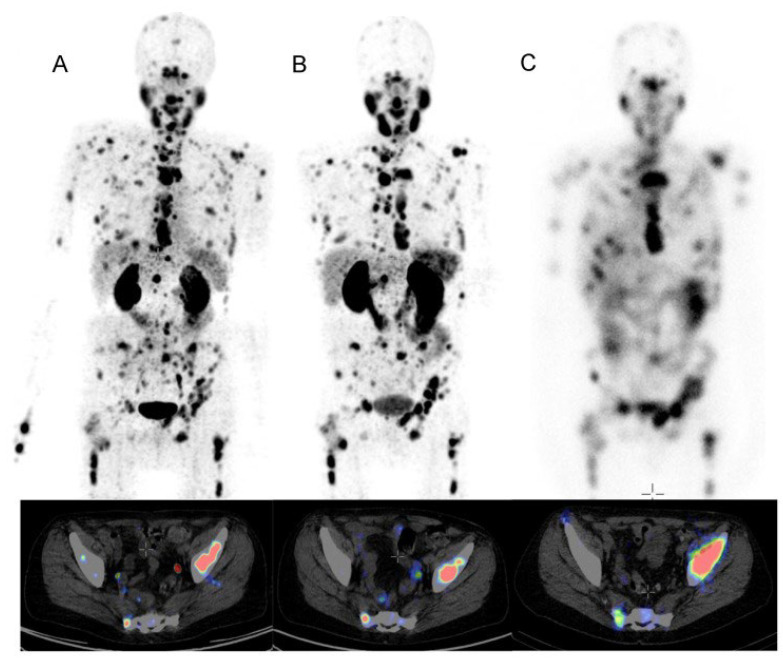
MIP (**top**) and slice (**bottom**) PET/CT of a 77-year-old mCRPC patient with high tumor load using (**A**) [^44^Sc]Sc-PSMA-617 (50 MBq, 60 min p.i.) and (**B**) [^68^Ga]Ga-PSMA-11 (120 MBq, 60 min p.i.); (**C**) planar scintigraphy (**top**) and SPECT/CT slice ~24 h after 6700 MBq [^177^Lu]Lu-PSMA-617 [32].

Clinical translation has been demonstrated across neuroendocrine and prostate cancer imaging. First-in-human studies using [^44^Sc]Sc-DOTATOC confirmed sensitive detection of metastatic neuroendocrine tumors without safety concerns [31]. In metastatic prostate cancer, [^44^Sc]Sc-PSMA-617 exhibited biodistribution comparable to ^68^Ga analogues, with favorable dosimetry characteristics [32] (Figure 5). ^44^Sc-labeled PSMA tracers serve as imaging surrogates for ^177^Lu-based radioligand therapy, facilitating individualized dosimetry paradigms [33]. This extended imaging window also supports centralized production and regional distribution models, which are not feasible with ^68^Ga, thereby enhancing the logistical scalability of ^44^Sc-based radiopharmaceuticals. Compared with [^68^Ga]Ga-PSMA-11 and [^68^Ga]Ga-DOTATOC, the longer physical half-life of ^44g^Sc allows later-time-point imaging (up to ~6 h post-injection), supporting improved tumor-to-background ratios for slowly accumulating lesions while preserving dosimetry performance that is broadly comparable to that of the reference ^68^Ga-based tracers [31,32,33].

Consistent with these in vivo observations, ^44g^Sc-labeled PSMA and somatostatin-targeted tracers retain receptor binding affinity and internalization kinetics comparable to their ^68^Ga and ^177^Lu analogues in LNCaP/PC-3 PIP and AR42J cell systems, as characterized in the accompanying in vitro evaluations of these first-in-human agents [31,32,33]. Dedicated phantom validation for ^44^Sc remains less extensively reported than for ^68^Ga; however, standardized PET/SPECT validation methodologies for novel radionuclides provide a practical framework for future cross-site harmonization of SUV quantification, scanner calibration, and radionuclide-specific corrections [34].

## 4. Manganese-52

Manganese-52 (^52^Mn; half-life = 5.59 d) is a positron-emitting radiometal (β^+^ = 29.6%, E_βave_ = 242 keV) whose multi-day half-life uniquely matches the biological kinetics of monoclonal antibodies, engineered proteins, liposomes, and nanomaterials, supporting immune-PET and longitudinal imaging [35]. The ^52^Mn decay scheme is provided in Figure 1.

### 4.1. Production

The preferred translational production route is ^52^Cr(p,n)^52^Mn using low- to mid-energy medical cyclotrons, operated at multiple academic centers in North America and Europe (including the University of Wisconsin, the University of Alabama at Birmingham, and European institutions within the PRISMAP network), which are currently the primary sources of research-grade ^52^Mn [35,36,37]. Optimized proton energies of approximately ~11–13 MeV maximize the (p,n) cross-section while limiting formation of long-lived ^54^Mn (t_½_ = 312 d), which arises predominantly at higher energies [35,36]. Another study reports the physical yields of ~10–15 MBq·µA^−1^·h^−1^ on enriched ^52^Cr under optimized low-energy proton irradiation conditions [37], with radionuclidic purities compatible with clinical translation when energy windows are carefully controlled. The principal practical limitations for ^52^Mn at present are (i) the high cost and limited commercial supply of enriched ^52^Cr target material, (ii) residual ^54^Mn impurity control at higher beam energies, and (iii) the absence of a generator system, which ties distribution to single-site cyclotron production [35,36,37]. The same ^52^Cr(p,n) and ^52^Cr(d,2n) reactions also co-populate the short-lived isomer ^52m^Mn (t_½_ = 21.1 min), which decays via β^+^ ~98% to stable ^52^Cr, along with only ~1.7% isomeric transition branch to ^52g^Mn ground state; the isomer therefore decays out completely during target processing and shipping, with negligible impact on the final radionuclidic profile. All subsequent references to ^52^Mn in this review denote the 5.59-day ground state (^52g^Mn) used for PET [35,36,37].

Electroplated ^52^Cr targets (99.89% radionuclidic purity) have become the preferred configuration for scalable production. The irradiation performance and post-irradiation 94% recovery of enriched chromium (replating efficiency: 60 ± 20%) was demonstrated [37], enabling recycling workflows that partially offset the cost of enriched target material.

Following irradiation, separation of ^52^Mn from bulk chromium is typically achieved via anion-exchange chromatography (e.g., AG1-X8) in hydrochloric acid media, exploiting the differential speciation of Mn and Cr chloride complexes; under optimized conditions, Cr^3+^ is efficiently washed from the column while ^52^Mn is retained and subsequently eluted in small volumes. Alternative multi-step purification workflows incorporating sequential anion- and cation-exchange have also been reported, enabling improved chromium decontamination, higher chemical purity, and enhanced recovery of enriched ^52^Cr for recycling [35,36,37]. The product shows high chemical purity with minimal metal contamination.

### 4.2. Radiolabeling

Unlike hard trivalent radiometals (e.g., Sc^3+^, Lu^3+^), Mn^2+^ is a borderline Lewis acid with high kinetic lability and accessible redox states (Mn^2+^/Mn^3+^), introducing additional complexity into chelator design. Stability therefore requires both thermodynamic affinity and kinetic inertness [38,39,40]. Using a cyclen-imidazole-based chelator, DOTI-Me was evaluated and showed that radiochemical conversion with ^52g^Mn was comparable to DOTA, with complex integrity assessed using DTPA challenge and serum-stability measurements [38]. Representative DOTA-based labeling of ^52^Mn is typically performed at pH 5–6 and ~90 °C for 30 min, with reported radiochemical yields > 95% and purities > 90% and apparent molar activities of the order of 1–10 MBq/nmol on peptide conjugates [40].

Rigid pyridinophane/bispidine-derived scaffolds highlight the importance of ligand preorganization for Mn(II) coordination. Pyridinophane-based tetraazacyclododecane derivatives, collectively referred to as the TE-series (TE-1, TE-5; where TE denotes the tetraaza-cyclododecane-ethyl scaffold), achieve quantitative ^52^Mn radiochemical conversion at 37 °C (60 min) and enable high apparent molar activity (especially with TE-1) labeling compatible with thermally sensitive biomolecules [41]. Chelation advances have enabled antibody constructs such as [^52^Mn]Mn-BPPA-trastuzumab, in which BPPA denotes the bispidine-based 6,6′-((6-((bis-pyridin-2-yl-methyl)-amino)pyridine-2,6-diyl)bis-(methylene))-dipicolinic-acid scaffold; the conjugate retains immunoreactivity and demonstrates HER2-specific tumor uptake with delayed imaging consistent with the ^52^Mn half-life. BPPA labeling is typically performed at pH ~6–7 and room temperature for 15–30 min, with radiochemical purity > 95% and apparent molar activities in the 5–15 MBq/nmol range preserving immunoreactivity [39,42].

More recently, the cyclohexyl-rigidified 18-membered dipyridyl-tetraazamacrocycle CHXPYAN has been introduced as a kinetically inert ^52^Mn(II) chelator; [^52^Mn]Mn-CHXPYAN remained >90% intact in mouse and human serum over 5 days and cleared rapidly from the liver and kidneys in vivo, whereas its flexible analogue PYAN retained an unchelated-Mn-like biodistribution similar to [^52^Mn]MnCl_2_. The reversible Mn(II)/Mn(III) electrochemistry of these macrocycles additionally opens the prospect of redox-responsive ^52^Mn radiopharmaceuticals [43].

Peptide-based radiopharmaceuticals have also been reported. One study [40] demonstrated successful labeling of DOTA-conjugated somatostatin analogs ([^52^Mn]Mn-DOTATATE and [^52^Mn]Mn-DOTA-JR11) under the conditions summarized above, confirming that macrocyclic scaffolds can accommodate Mn^2+^ while maintaining receptor targeting.

Beyond chelation, ^52^Mn can be incorporated into manganese-based nanostructures such as [^52^Mn]MnO_2_ and [^52^Mn]Mn_3_O_4_ via lattice or surface coordination [44]. In H_2_O_2_ and glutathione-rich tumor environments, [^52^Mn]MnO_2_ undergoes redox conversion to Mn^2+^, producing oxygen and reactive oxygen species through Fenton-like mechanisms, with stability governed by crystal phase and surface engineering [44].

Overall, BPPA and pyridinophane/TE-series (together with preorganized bispidine-type scaffolds) and PYAN (CHXPYAN)-based chelators currently appear best suited for immune-PET-oriented ^52^Mn applications because they tolerate mild labeling conditions and preserve immunoreactivity, whereas DOTA remains a pragmatic choice for heat-stable peptide vectors such as DOTATATE and DOTA-JR11 [38,39,40,41,42]. For nano-construct-based applications, direct lattice incorporation into MnO_x_ platforms is the most practical route at present [44].

### 4.3. Translational Applications

**Figure 6:**
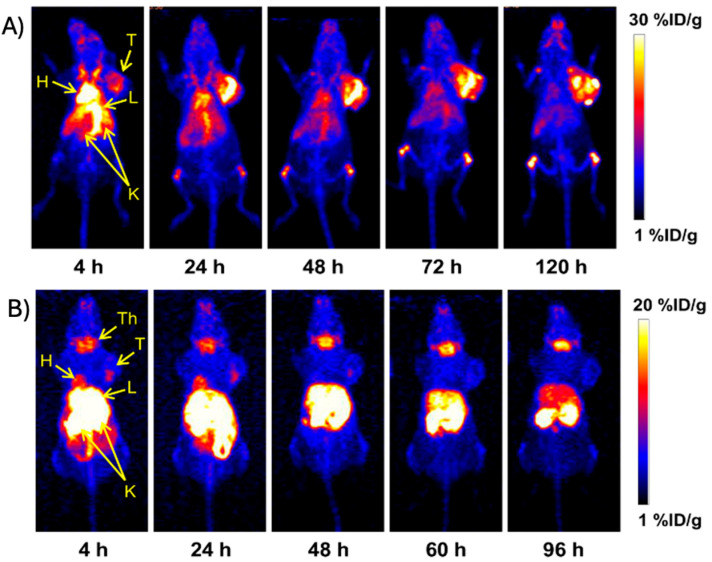
Serial MIP PET of mice with [^52^Mn]Mn-DOTA-TRC105 (**A**) and [^52^Mn]MnCl_2_ (**B**): thyroid uptake in [^52^Mn]MnCl_2_ but not [^52^Mn]Mn-DOTA-TRC105 confirms stable DOTA chelation. H = Heart; L = Liver; K = Kidneys; T = Tumor; Th = Thyroid [45].

In vitro, ^52^Mn-labeled antibody and peptide constructs demonstrate retained receptor binding and immunoreactivity, for example, [^52^Mn]Mn-BPPA-trastuzumab retaining HER2-specific binding showed selective uptake in HER2-positive xenografts, and [^52^Mn]Mn-DOTATATE and [^52^Mn]Mn-DOTA-JR11 retain SSTR2-specific binding and internalization patterns consistent with agonist/antagonist behavior reported for established SSTR-targeted tracers [39,40,41,42]. These in vitro data support the in vivo observations below and highlight functional equivalence with established HER2- and SSTR-targeted radiopharmaceuticals. The importance of chelator selection is illustrated in vivo by the absence of thyroid uptake with [^52^Mn]Mn-DOTA-TRC105 in contrast to free [^52^Mn]MnCl_2_, supporting substantial in vivo stability of the DOTA complex over the imaging window (Figure 6).

The physical decay properties of ^52^Mn (β^+^ = 29.6%) and multi-day half-life allow studies of slow biological processes supporting longitudinal imaging with favorable spatial resolution relative to longer-lived PET isotopes [45]. HER2-targeted tracers such as [^52^Mn]Mn-BPPA-trastuzumab demonstrate specific tumor localization and sustained imaging over multiple days [40,41], exceeding the imaging window achievable with [^68^Ga]Ga-DOTA-antibody constructs and complementing the biodistribution profiles available with [^89^Zr]Zr-trastuzumab while providing a long-lived β^+^ PET alternative for biologics on a days-scale imaging axis. A recent comparative PET evaluation of [^52^Mn]Mn-DOTATATE (agonist) and [^52^Mn]Mn-DOTA-JR11 (antagonist) has further characterized SSTR2-targeted imaging with ^52^Mn in vitro and in vivo [40]. Radiomanganese PET has also been applied to imaging pancreatic β-cell mass in diabetes models, exploiting manganese’s role as a calcium analogue entering active β-cells via voltage-gated calcium channels [46].

Neuroimaging studies further demonstrate the capacity of ^52^Mn to trace neuronal pathways, although dose-dependent manganese accumulation requires careful toxicity evaluation [47]. While the positron characteristics are favorable, ^52^Mn emits higher-energy γ-photons (e.g., 744, 936, 1434 keV), contributing to additional patient dose. Dosimetry modeling and administered-activity optimization are therefore required for clinical translation [45]. Because administered activities for ^52^Mn immune-PET are likely to be lower than those used for routine ^68^Ga or ^18^F PET, phantom-based cross-calibration and prompt-γ correction remain important translational deliverables, even though dedicated human phantom studies for ^52^Mn remain limited at present [45].

## 5. Lead-203

Lead-203 (^203^Pb; t_½_ = 51.9 h) decays exclusively by electron capture to stable ^203^Tl, with a single dominant γ emission at 279.2 keV (intensity 80.9%), well matched to the energy window of clinical SPECT/CT cameras and no β^−^ contribution. These properties make ^203^Pb the diagnostic partner of choice for ^212^Pb in image-guided targeted alpha therapy (the decay scheme is provided in Figure 1). ^203^Pb SPECT/CT enables patient-specific pharmacokinetics and dosimetry to guide ^212^Pb therapy, with studies evaluating gamma-camera imaging characteristics of the ^203/212^Pb theranostic pair [48]. First-in-human imaging with [^203^Pb]Pb-VMT-α-NET demonstrated tumor-site uptake concordant with [^68^Ga]Ga-DOTANOC in metastatic neuroendocrine tumors (NETs) [49], while ligand-optimization studies continue to improve the paired ^203/212^Pb peptides [50].

### 5.1. Production

The predominant clinical production route for ^203^Pb described in recent optimization studies is proton irradiation of electroplated ^205^Tl targets via the ^205^Tl(p,3n)^203^Pb reaction at ~24 MeV on medical cyclotrons [51]. This route is currently operated at a small number of North American and European centers (including the University of Alabama at Birmingham, the University of Iowa, and European facilities affiliated with the PRISMAP network) that can accommodate 24 MeV proton beams and the associated solid-target infrastructure [51,52]. Reported thick-target yields are of the order of hundreds of MBq per μA·h, sufficient for multi-patient imaging after single-irradiation workflows [51]. Electroplated targets (typically 76–114 mg/cm^2^) are prepared on copper or gold backings to ensure mechanical stability and heat dissipation during irradiation [51]. Post-irradiation, the target is dissolved in nitric acid and purified using a two-column separation method involving extraction resin (PbCl_4_^2−^ AG-type) followed by weak cation exchange, with final elution of [^203^Pb]PbCl_2_ in 1 M HCl suitable for radiolabeling [51]. Despite these optimizations [51,52], access to enriched ^205^Tl, the cost of enriched target material, and the limited number of cyclotrons capable of 24 MeV proton irradiation remain major barriers for broader deployment [51,52,53].

Generator-based approaches are at an early stage, with an ongoing exploration ^203^Pb (t_½_ = 51.9 h) production from short-lived ^203^Bi (t_½_ = 11.8 h). Recent work demonstrated cyclotron production of ^204^Bi via 24 MeV proton irradiation of natural Pb foils, followed by rapid oxidative dissolution (3 M HNO_3_/30% H_2_O_2_), chloride matrix conversion, and anion-exchange processing to establish a ^204^Bi/^204m^Pb generator system with co-produced ^203^Bi, enabling ingrowth and elution of ^203/204m^Pb [54]. To date, no commercial ^203^Pb generator exists, and current clinical/preclinical supply relies on cyclotron production from ^203/205^Tl targets. In summary, the principal translational constraints for ^203^Pb are (i) accessibility of enriched ^203/205^Tl target material, (ii) the limited global availability of 24 MeV-class medical cyclotrons, and (iii) the absence of a commercial generator, collectively restricting routine use to a relatively small number of centers despite the radiopharmaceutical maturity demonstrated in first-in-human studies [51,52,53,54]. Recent advances in dosimetry and imaging for the ^203^Pb/^212^Pb theranostic pair have been summarized elsewhere and provide a framework for clinical translation [55].

### 5.2. Radiolabeling

Radiolabeling for the ^203/212^Pb theranostic pair has traditionally relied on macrocyclic chelators such as DOTA and the tetra-acetamide derivative 1,4,7,10-tetrakis(carbamoylmethyl)-1,4,7,10-tetraazacyclododecane (TCMC, also referred to as DOTAM), which stabilizes Pb^2+^ and limits in vivo metal dissociation and daughter redistribution [56,57,58]. Due to its larger ionic radius and stereochemically active lone pair, Pb^2+^ favors hemidirected coordination geometries that can reduce kinetic inertness unless tightly encapsulated [56,57]. Representative DOTA and TCMC labeling of ^203^Pb is performed at pH 5–7 (ammonium acetate buffer) and 37 °C (or room temperature) for 30–60 min, routinely yielding apparent molar activities of 10–50 MBq/nmol, radiochemical purities ≥ 95%, and overall labeling efficiencies > 90% after solid-phase cleanup [57,58].

A recently reported Pb-specific chelator (PSC) forms a neutral Pb^2+^ complex and enables rapid radiolabeling under mild conditions [56]. Labeling is typically performed at pH ~5.5–6 in acetate buffer at room temperature, whereas TCMC and DOTA labeling occurs at pH 5–6.5 and pH 5–7, respectively, under buffered conditions [57,58]. These studies highlight the advantages of TCMC and Pb-specific chelators in minimizing thermal stress while maintaining high in vitro stability.

For [^203^Pb]Pb-PSC-panitumumab, the radiolabeling was performed at pH ~5.0 using [^203^Pb]Pb(OAc)_2_ at room temperature for 5–10 min, achieving >99% incorporation by radio-TLC, the isolated radiochemical yield was 41.4 ± 8%, with molar activity reported as 1.2 ± 0.35 GBq/mg [59]. The purified immunoconjugate retained antigen binding and enabled in vivo SPECT/CT imaging of EGFR-expressing xenografts, demonstrating stability of the Pb-PSC complex [59]. Purification typically involves column-based cleanup to remove unbound lead, while reaction optimization requires mildly acidic conditions (pH 5–6) consistent with Pb^2+^ hydrolysis behavior [56].

Beyond solution-phase labeling, resin-appended 18-membered macrocycles enable selective rare-earth (Ln^3+^) capture and separation [60]. Although demonstrated for lanthanides rather than Pb^2+^, these immobilized macrocycles suggest potential capture–release platforms for radiometals [60].

On the basis of current evidence, TCMC (DOTAM) is recommended as the most established chelator for ^203^/^212^Pb theranostic applications because it provides high in vivo stability, an improved retention of daughter radionuclides after decay, and is already used in clinical-stage ^212^Pb constructs whereas PSC is the preferred option for heat-sensitive biologics such as antibodies because it supports room-temperature labeling while maintaining comparable stability [56,57,58,59].

### 5.3. Translational Applications

In vitro, [^203^Pb]Pb-PSC-panitumumab retains EGFR-specific binding affinity and demonstrates specific tumor uptake and retention in patient-derived EGFR-positive head-and-neck squamous cell carcinoma cells, consistent with the stability of the Pb–PSC complex characterized in prior radiolabeling studies [59]. Analogous retained receptor-binding has been described for [^203^Pb]Pb-VMT-α-NET, which binds SSTR2 with affinity comparable to [^68^Ga]Ga-DOTATATE and [^177^Lu]Lu-DOTATATE in preclinical cell systems [61]. These in vitro profiles support preserved target-specific binding and biodistribution comparable to established reference agents and support the in vivo findings below.

A PSMA ligand suitable for labeling with lead radioisotopes was developed to evaluate dosimetry for simulated ^212^Pb-based α-therapy using ^203^Pb as an imaging surrogate. For completeness, ^212^Pb (t_½_ = 10.6 h) is a pure β^−^ emitter (Eβ^−^,max ≈ 0.57 MeV) decaying to ^212^Bi (t_½_ = 60.6 min); ^212^Bi subsequently emits either an α-particle (Eα = 6.05/6.09 MeV; 36%) directly to ^208^Tl, or a β^−^ (64%) to ^212^Po (t_½_ = 0.30 µs) which immediately α-decays (Eα = 8.78 MeV) to stable ^208^Pb. Each ^212^Pb decay therefore delivers, on average, one α-particle to the target site. Imaging of two metastatic prostate cancer patients demonstrated that [^203^Pb]Pb-CA012 produced adequate γ-ray emission for planar imaging at injected activities of 250–300 MBq. However, lower count rates than [^177^Lu]Lu-PSMA-617 limited practical SPECT acquisition times [62].

Building on this approach, a feasibility study in a patient with end-stage midgut neuroendocrine tumor refractory to [^177^Lu]Lu-HA-DOTATATE therapy utilized 224 MBq of [^203^Pb]Pb-VMT-α-NET for planar and 22 h SPECT/CT imaging. Comparison with [^68^Ga]Ga-HA-DOTATATE PET/CT revealed high [^203^Pb]Pb-VMT-α-NET uptake in liver metastases, consistent with PET findings [49].

Similar concordance was found in an additional patient imaged with [^203^Pb]Pb-VMT-α-NET, a somatostatin receptor-targeting agent, and [^68^Ga]Ga-DOTANOC [61]. Building on these proof-of-concept observations, a subsequent multi-patient series reported the first clinical experience with the [^203/212^Pb]Pb-VMT-α-NET theranostic agent in progressive metastatic gastroenteric-pancreatic NETs [63]. The stability and decay properties of lead isotopes, PSMA targeting, and reduced radiation burden of ^203^Pb imaging support further clinical evaluation of this theranostic strategy. Phantom studies characterizing γ-camera performance for the ^203/212^Pb pair have also been reported [48] and provide a framework for harmonized SPECT/CT acquisition, which will be particularly important as ^203^Pb-based theranostic workflows expand into multicenter clinical trials. A dedicated Phase 0 imaging trial of [^203^Pb]Pb-VMT-α-NET demonstrated its utility for dosimetry and treatment planning, with higher sensitivity for lesions >1 cm than for smaller or non-measurable lesions [64]. These trials are part of a broader clinical experience with targeted α-emitter peptide-receptor radionuclide therapy (α-PRRT) in SSTR-positive NETs [65].

## 6. Cobalt-55

Cobalt-55 (^55^Co) is a positron-emitting radiometal with a half-life of 17.5 h, enabling centralized production, quality control, and regional distribution while supporting imaging of slower-clearing vectors such as peptides and antibody fragments [66]. It exhibits a high β^+^ branching ratio (77%) and a prominent γ-emission at 931 keV (~75%), characteristics that distinguish it from pure β^+^-emitters and require appropriate correction considerations in quantitative PET imaging [66] (decay scheme in Figure 1). The γ-signature of ^55^Co has motivated interest in emerging multiplexed (“triplexed”) PET to separate a ^55^Co-labeled tracer from a pure β^+^ tracer within a single session, underscoring the need for radionuclide-specific correction algorithms to account for prompt γ-emissions and cascade coincidences.

### 6.1. Production

The established production routes include proton irradiation of enriched ^58^Ni targets through the ^58^Ni(p,α)^55^Co reaction at widely available 16–18 MeV and low-energy (~13–16 MeV) medical cyclotrons, a configuration that is currently operated at several centers in Europe and North America (including the University of Wisconsin–Madison, the University of Alabama at Birmingham, Odense University Hospital, and Lund University Hospital), which are the reported centers to produce research-grade ^55^Co [66,67]. In facilities equipped with deuteron beams, enriched ^54^Fe targets may alternatively be irradiated within an optimized 5–8 MeV energy window to enable efficient production [66,67]. This ^54^Fe(d,n)^55^Co pathway provides high radionuclidic purity (99.995% at EOB) when enriched iron targets are used while the ^58^Ni(p,α)^55^Co route yields ~98.8% radionuclidic purity under optimized conditions [67]. For ^58^Ni(p,α)^55^Co, excitation functions and stacked-foil measurements indicate that proton energies are selected to minimize long-lived impurities, particularly ^57^Co from the ^58^Ni(p,2p) reaction, which becomes accessible above ~15 MeV [68]. Thus, practical operating windows are chosen to balance ^55^Co yield while suppressing higher-energy side reactions [66,68]. For ^54^Fe(d,n), the cross-section peaks near ~6–7 MeV, and practical stacked-foil irradiations are conducted around ~8 MeV degraded through the peak region, which limits competing channels and supports high radionuclidic purity [67,68]. These energy windows and impurity thresholds are consistent with experimental excitation-function data and nuclear-data evaluations [67,68]. Using enriched targets, reported thick-target yields under routine medical cyclotron conditions are ~9–10 MBq·µA^−1^·h^−1^ for both ^58^Ni(p,α) and ^54^Fe(d,n) routes under optimized energy control, which is sufficient to support regional distribution given the 17.5 h half-life [67]. Variations from theoretical expectations are attributed to beam–target area mismatch, electroplating quality, and insufficient thermal dissipation from target [67]. The principal practical limitations for ^55^Co are (i) the cost and availability of enriched ^58^Ni or ^54^Fe target material, (ii) tight control of ^57^Co/^56^Co long-lived impurity channels, and (iii) the absence of a commercial generator system [66,67,68]. Few dedicated reviews on radiocobalt theranostic applications provide an updated landscape of production, chelation chemistry, and translational prospects for ^55^Co and ^58m^Co [66,67,68,69].

Enriched ^58^Ni remains the predominant target for ^55^Co production due to compatibility with widely available ~10–15 MeV medical cyclotrons and established HCl-based dissolution followed by DGA-resin extraction chromatography for Co/Ni separation [67]. Pressed ^58^Ni/Mg targets enable >200 MBq ^55^Co production with rapid room-temperature dissolution (~10 min) and streamlined Co/Ni separation (~1–1.5 h), improving production throughput and integration with labeling and QC workflows [70].

### 6.2. Radiolabeling

Chelation studies indicate that cage chelators such as DiAmSar (DSar) forms kinetically inert ^55^Co complexes, with optimized labeling at pH ~8 and 37–80 °C, apparent molar activity increased with temperature reaching 45 ± 9 MBq/nmol at 80 °C after 4 h, together with strong serum/EDTA stability (≥95% intact) and favorable mouse pharmacokinetics [71]. In parallel, head-to-head work with GRPR-targeted RM26 analogs demonstrated efficient radiocobalt labeling across NOTA, NODAGA, DOTA, and DOTAGA under comparable conditions (pH 5–6, 40–95 °C, 15–30 min), with radiochemical yields > 90% and enabling chelator ranking based on receptor binding, serum stability, and biodistribution [72]. In these systems, chelator effects were construct-dependent: DOTA-PEG_2_-RM26 showed the strongest tumor retention and tumor-to-blood ratios, whereas NOTA-containing analogues showed favorable renal clearance consistent with their coordination properties [72].

^55^Co vector development has expanded beyond GRPR to include neurotensin receptor–1 (NTSR1)-targeted peptides. In the NTSR1 system NT-CB-NOTA, ^55^Co labeling achieved high radiochemical purity and apparent molar activity comparable to ^64^Cu and ^68^Ga analogues under matched conditions [73]. These tracer studies emphasize a consistent pattern: ligands that enforce higher kinetic inertness and accommodate Co(III) oxidation deliver better in vivo stability and image contrast [72].

Radiocobalt chemistry is also exploring new vector classes. A polypyridylamine (TPENYNE) chelate, conjugated via click chemistry to azide-Peg_2_-linker of EGFR-targeting peptide GE11, was labeled with ^57^Co, as a surrogate for ^55/58m^Co, supporting radiochemical feasibility for ^55^Co translation but demonstrating poor in vivo tumor targeting, thereby limiting its suitability as an EGFR-directed radiocobalt construct [74]. Together with growing theranostic interest in the ^55^Co/^58m^Co pair, including preclinical evaluation of [^58m^Co]Co-DOTA-PSMA-617 Auger therapy and PET/CT imaging of [^55^Co]Co-DOTA-PSMA-617, these advances are supporting the continued development of element-matched cobalt theranostic pairs [74,75] and are echoed in recent reports [66].

On the basis of available comparative data, NOTA/NODAGA and cross-bridge macrocycles (including DiAmSar and NT-CB-NOTA) are recommended as the preferred chelators for ^55^Co peptide tracers because they combine mild labeling conditions with the highest in vivo stability [71,72,73], while DOTA remains a practical option for DOTA-platform peptides and for ^58m^Co Auger constructs where Auger-emitter stability and intracellular retention are relevant [74,75]. Because NOTA, NODAGA and DiAmSar were all originally developed for Cu^2+^ coordination, ^64^Cu, which is far more widely available than ^55^Co, can in principle serve as a chemically equivalent imaging and dosimetry surrogate for ^58m^Co-based Auger therapy, an attractive route given the limited current supply of ^55^Co [66].

### 6.3. Translational Applications

In vitro, ^55^Co-labeled NT-CB-NOTA demonstrates NTSR1-specific binding and tumor uptake consistent with its ^64^Cu- and ^68^Ga-labeled analogues under matched conditions [73], and [^55^Co]Co-DOTA-PSMA-617 retains PSMA-specific binding in LNCaP cells broadly comparable to [^68^Ga]Ga-PSMA-617 and [^177^Lu]Lu-PSMA-617 reference tracers [74,75]. These data indicate that ^55^Co-based constructs retain target-specific binding and biodistribution consistent with established reference radiopharmaceuticals in preclinical cell systems.

**Figure 7:**
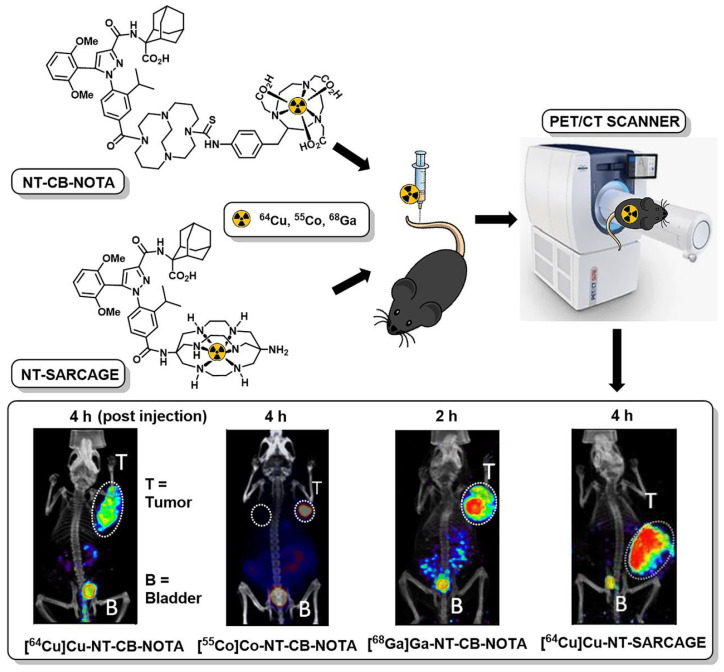
Representative in vivo PET/CT imaging at 4 h post-injection using [^64^Cu]Cu-NT-CB-NOTA, [^55^Co]Co-NT-CB-NOTA, [^68^Ga]Ga-NT-CB-NOTA, and [^64^Cu]Cu-NT-Sarcage radiopharmaceuticals targeting NTSR-1-positive cancers [73].

Due to its half-life and distinct decay properties, ^55^Co has attracted increasing preclinical and exploratory clinical interest for diagnostic PET imaging and theranostic application [66,71]. Preclinical in vivo studies have focused on peptides and small molecules targeting tumor-specific receptors. For example, ^55^Co- labeled albumin-binding folate derivatives showed high tumor uptake and good stability in mouse models of folate receptor-expressing ovarian and lung cancers [66]. Comparative imaging with [^64^Cu]Cu- and [^68^Ga]Ga-labeled analogues indicates that ^55^Co can deliver tumor-to-background ratios that are comparable to, and in some cases improved relative to these reference radiometals, especially at late imaging time points where the 17.5 h half-life provides longer biodistribution windows [66,73]. Comparative PET/CT at 4 h post-injection demonstrates that [^55^Co]Co-NT-CB-NOTA achieves tumor contrast comparable to its [^64^Cu]- and [^68^Ga]-labeled counterparts in NTSR-1-positive xenografts, supporting ^55^Co as a viable PET surrogate for matched-pair theranostics (Figure 7) [66,73].

Clinically, ^55^Co in PET imaging has been explored for imaging of cerebrovascular disease. Comparative investigations of ^55^Co uptake with cerebral blood flow, oxygen metabolism, blood volume, and structural imaging parameters have shown that tracer accumulation in ischemic stroke correlates with perfusion deficits and metabolic impairment, suggesting potential sensitivity to ischemic tissue changes. These findings further suggest that PET imaging with ^55^Co reflects persistent metabolic patterns associated with ischemic damage [76]. Dedicated phantom studies for ^55^Co remain limited, but quantitative benchmarking against ^68^Ga and ^18^F phantoms remains an important area for future validation that will be essential to support routine clinical use, particularly given the prompt 931 keV γ-emission and associated requirement for prompt-γ correction modeling [66,68].

## 7. Development Imperatives and Conclusions

Clinical translation of the radiometals considered here depends on continued progress in isotope production, radiochemistry, and quantitative imaging frameworks. Reliable supply requires access to enriched target materials and scalable production routes, including ^58^Ni(p,α)^55^Co, ^52^Cr(p,n)^52^Mn, ^205^Tl(p,3n)^203^Pb, ^44^Ca(p,n)^44g^Sc, and ISOL-based ^149^Tb, supported by solid-target processing, recycling workflows, and automated purification. Expansion of production infrastructure and harmonized regulatory pathways will support clinical availability. The decay characteristics, optimal production routes, beam-energy windows, and radiochemistry features of the five emerging radiometals discussed in this review are summarized in Table 1.

Across the five radionuclides, production feasibility varies significantly: ^44^Sc and ^203^Pb are the most accessible using medical cyclotrons, whereas ^52^Mn and ^55^Co require enriched targets with tighter impurity control, and ^149^Tb remains restricted to ISOL-based facilities. This gradient in production complexity is a key determinant of their translational readiness and clinical scalability. A consolidated QA (quality assurance)/QC (quality control) checklist covering radionuclidic purity, chelation efficiency, and release criteria for ^149^Tb, ^44^Sc, ^52^Mn, ^203^Pb, and ^55^Co is provided in Table 2 to support harmonized translational workflows.

**Figure 8:**
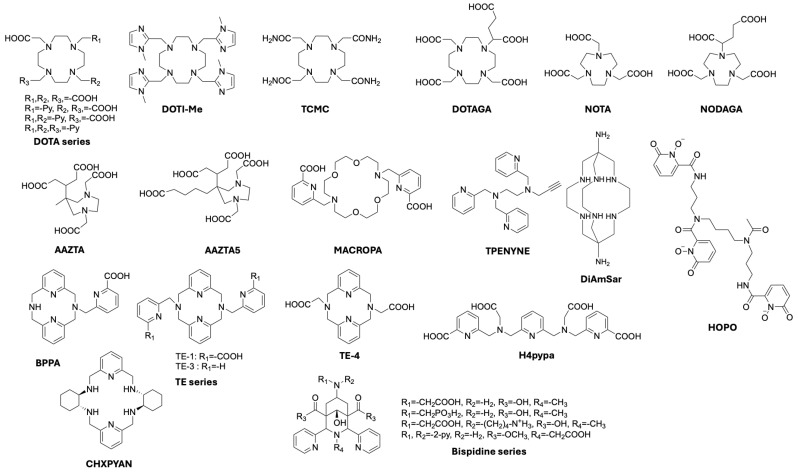
Structures of chelators mentioned in Table 3.

Advances in chelation chemistry remain essential for translation, as metal-specific ligand systems are required to achieve the kinetic inertness and in vivo stability across Mn^2+^, Co^2+^, Pb^2+^, and hard trivalent metals (Tb^3+^, Sc^3+^) coordination systems [77]. Table 3 maps each radiometal to its compatible chelator families (e.g., DOTA, TCMC, etc.; Figure 8), highlighting the metal-specific coordination preferences. Radiochemically, clear distinctions emerge across these radiometals: hard trivalent metals (^149^Tb, ^44^Sc) are well served by DOTA-based coordination chemistry, whereas transition metals (^52^Mn, ^55^Co) require more specialized ligand architectures to address redox activity and kinetic lability. In contrast, Pb^2+^ demands chelators such as TCMC that accommodate its larger ionic radius and stereochemically active lone pair, underscoring the necessity of metal-specific chelator design rather than a universal coordination approach. Improved radiochemical processing, including resin-based separations and modular conjugation, enables reliable preparation of complex biomolecular vectors.

Quantitative imaging considerations are equally central to translation. As summarized in the production and imaging frameworks presented herein (Table 4), radionuclidic purity, excitation-function optimization, impurity control, and validated scanner-specific corrections are required for accurate quantification. Prompt-γ co-emissions, β^+^ branching variability, and long-lived impurity channels affect PET performance and therefore require validated dead-time modeling, activity-dependent calibration, and harmonized acquisition protocols to ensure reproducible dosimetry estimation across institutions.

Collectively, these factors define the pathway from isotope production to clinical implementation, highlighting that successful translation requires coordinated advances in production scalability, radiochemistry optimization, and quantitative imaging standardization.

### 7.1. Pharmaceutical Readiness of the Five Radiometals

Based on the available literature, the five radiometals reviewed here fall into three broadly defined stages of pharmaceutical readiness. ^203^Pb is the most clinically advanced, with first-in-human SPECT/CT imaging of [^203^Pb]Pb-CA012 and [^203^Pb]Pb-VMT-α-NET already reported and an established role as the imaging surrogate for ^212^Pb-based α-therapy, supported by a mature chelator toolkit (DOTA/TCMC/PSC) and defined production routes [48,49,50,51,52,54,56,57,58,59,60,61,62]. ^44g^Sc is in early clinical imaging use (first-in-human [^44^Sc]Sc-DOTATOC and [^44^Sc]Sc-PSMA-617), with dosimetry performance comparable to ^68^Ga analogues and clinically actionable theranostic pairing with ^177^Lu and is therefore well positioned as the next-closest candidate to routine clinical use [31,32,33]. ^149^Tb, ^52^Mn, and ^55^Co are currently at the preclinical-to-exploratory-clinical stage: ^149^Tb and ^55^Co have demonstrated human imaging feasibility in limited patient series (^149^Tb via β^+^-PET and ^55^Co in stroke imaging) and strong preclinical therapeutic and imaging performance, while ^52^Mn remains primarily a preclinical immune-PET and neuroimaging tool with multi-day biodistribution windows [5,12,45,46,47,66,76].

### 7.2. Potential Alternatives to Currently Used Radiometals

Within this framework, ^44g^Sc represents the most compelling near-term alternative to ^68^Ga for PSMA- and somatostatin-receptor PET imaging, particularly when extended imaging windows or compatibility with ^177^Lu dosimetry is required [31,32,33]. ^203^Pb is already positioned as the diagnostic component of the ^212^Pb/^212^Bi theranostic pair, offering a robust SPECT surrogate for α-particle therapy [48,49,50,51,52,54,56,57,58,59,60,61,62]. ^52^Mn provides a credible alternative to ^89^Zr for days-scale immune-PET of antibody and nano-construct vectors, with the caveat that β^+^ branching is lower [40,41,42,43,44,45]. ^55^Co is most compelling in element-matched pairing with Auger-emitting ^58m^Co, enabling an integrated diagnostic–therapeutic platform that is not yet routinely available with other PET radiometals [66,74,75]. ^149^Tb is unique in that it simultaneously delivers α-therapy and PET imaging within a single radionuclide, complementing rather than replacing ^225^Ac/^213^Bi-based α-therapy [5,12,13,18].

### 7.3. Practical Limitations

Each of these radiometals also faces clearly identifiable practical barriers to routine clinical translation [13,53,70,78,79], analogous to the supply [80] and infrastructure challenges that also limit established α-emitters such as ^225^Ac and ^213^Bi [81]. ^149^Tb is the most constrained, requiring GeV-class proton accelerators, ISOL-based mass separation, and specialized lanthanide radiochemistry (CERN-MEDICIS/PSI) to reach research-grade supply; α-recoil-induced redistribution of long-lived daughters is an additional dosimetry consideration [5,7,10]. On another note, two commonly proposed mitigation strategies warrant brief comment. The first, namely upgrading a conventional ~16 MeV medical cyclotron through higher proton extraction energies or auxiliary charged-particle beam capabilities, is not a straightforward retrofit because such systems are fundamentally constrained by fixed magnet geometry, extraction radius, shielding design, and beamline architecture. In practical terms, routine implementation of direct ^149^Tb production routes would require a substantially different accelerator configuration capable of supporting reactions such as ^152^Gd(p,4n)^149^Tb, which has been investigated experimentally over the ~30–66 MeV proton energy window with a cross-section maximum of ~250 mb near 42 MeV, or alternative light-charged-particle routes including ^151^Eu(^3^He,5n)^149^Tb (cross-section ~70 mb at ~47 MeV) [82,83,84].

**Table 1:** Summary of key properties of ^149^Tb, ^44^Sc, ^52^Mn, ^203^Pb, and ^55^Co, including production routes with beam-energy windows and radiochemistry characteristics.

Isotope	Half-Life (h)	Positron Decay (%)	Cyclotron Production (Reaction → Typical Beam Energy Window)/Generator Production	Dosimetry Readiness/Translation Stage	References
Tb-149	~4.12	~7.1	^152^Gd(p,4n)^149^Tb → ~45–60 MeV p	Preclinical (in vivo therapeutic models)	[5,6,12,13]
			^151^Eu(3He,5n)^149^Tb → ~35–50 MeV 3He		
			(ISOL: proton spallation on Ta/W + on-line mass separation; facility-specific GeV p)		
Sc-44	~4.04	~94	^44^Ca(p,n)^44g^Sc → ~9–13 MeV p (minimize ^44m^Sc)	First-in-human imaging; early clinical dosimetry	[23,31,79]
			^44^Ca(d,2n)^44^Sc → ~14–19 MeV d		
			^44^Ti → ^44^Sc (generator)		
Mn-52	~134.16	~29–30	^52^Cr(p,n)^52^Mn → ~11–13 MeV p (enriched ^52^Cr) or at ~12–18 MeV p (enriched ^52^Cr, with ^54^Mn impurity management)	Preclinical (antibody and small animal imaging)	[39,45,77]
Pb-203	~51.9	0 (EC; SPECT surrogate for ^212^Pb)	^203^Tl(p,n)^203^Pb → ~11–18 MeV p	First-in-human SPECT imaging; clinical theranostic pairing	[48,49,56]
			^205^Tl(p,3n)^203^Pb → ~24–30 MeV p		
			^nat^Pb(p,xn)^203^Bi → EC → ^203^Pb → ~14–22 MeV p		
			^206^Pb(p,4n)^203^Bi → EC → ^203^Pb → ~30–40 MeV p		
Co-55	~17.5	~77	^58^Ni(p,α)^55^Co → ~13–16 MeV p (pressed ^58^Ni targets)	Preclinical; limited exploratory human data	[70,73,76,78]
			^54^Fe(d,n)^55^Co → ~7–10 MeV d		
			^56^Fe(p,2n)^55^Co → ~18–28 MeV p (with ^56/57^Co impurity control)		

**Table 2:** QA/QC checklist for translation of ^149^Tb, ^44^Sc, ^52^Mn, ^203^Pb, and ^55^Co.

Category	Translation Parameter	In Relevance to Tb-149, Sc-44, Mn-52, Pb-203, Co-55	References
Radionuclidic Control	Long-lived impurity suppression	Critical for ^52^Mn (^54^Mn), ^55^Co (^56/57^Co), ^44^Sc (^44m^Sc), ^203^Pb (^202/201^Pb)	[39,49,70,79]
	Excitation-function energy window validation	Required to prevent impurity channels (Ni, Fe, Cr, Tl targets)	[39,49,79]
	Isomeric purity (where applicable)	Important for ^44^Sc/^44m^Sc discrimination	[23,79]
Target-Material Specifications	Enriched target isotopic composition certification	^58^Ni, ^54^Fe, ^44^Ca, ^203/205^Tl, ^52^Cr	[39,48,70,79]
	Impurity accumulation during recycling	Particularly relevant for Ni, Fe, Cr systems	[39,70]
	Target integrity under irradiation	Pressed Ni/Mg and electroplated targets require thermal validation	[70]
Post-Irradiation Chemical Separation	Reproducible metal separation yield	Co/Ni, Mn/Cr, Pb/Tl separation robustness	[10,39,49,70]
	Trace metal carryover (target metal)	Residual target-metal ions Ni^2+^, Fe^3+^, Cr^3+^, Tl^+^ reduce molar activity	[49,70,79]
	Compatibility with automation modules	Needed for clinical translation of solid-target isotopes	[70,79]
Chelation-Specific Considerations	Metal oxidation state control	^55^Co (Co^2+^/Co^3+^), ^52^Mn (Mn^2+^/Mn^3+^) redox management	[41,56,71]
	Kinetic inertness validation	Particularly important for Mn^2+^ and Co^2+^ complexes	[41,56,71,77]
	Molar activity reproducibility	Sensitive to trace metal contamination	[23,79]
Generator-Specific Considerations	Parent breakthrough monitoring	^44^Ti in ^44^Ti/^44^Sc systems	[23,79]
	Elution profile reproducibility	For generator-based ^44^Sc workflows	[23,79]
Imaging-Specific Validation	Prompt-γ correction modeling	Relevant for quantitative PET imaging with ^55^Co, ^52^Mn, ^44^Sc due to prompt γ-emissions	[45,73,78,79]
	Low β^+^ branching quantification strategy	Relevant for PET sensitivity in case of ^149^Tb	[12]
	Dosimetry modeling readiness	Needed for ^203^Pb as ^212^Pb surrogate and long-lived ^52^Mn studies	[48,56]
Process Robustness	Batch-to-batch radionuclide consistency	Required for multicenter translation	[49,70,79]
	Standardized excitation-window reporting	Improves cross-site reproducibility	[39,49,79]

**Table 3:** Chelation compatibility for ^149^Tb, ^44^Sc, ^52^Mn, ^203^Pb, and ^55^Co (chelator compatibility is supported by published radiolabeling data and by established coordination chemistry principles (ionic radius, hardness, coordination number) when direct isotope-specific evidence is limited).

Isotope	Chelators	References
Tb-149	DOTA family	[5,18,77]
	MACROPA	MACROPA and expanded-cavity macrocycles were developed primarily for large trivalent ions (e.g., Ac^3+^); while chemically plausible for ^149^Tb due to Tb^3+^ ionic radius and coordination chemistry, further labeling validation is required; [56]
Sc-44	DOTA	[56,79]
	AAZTA/AAZTA5	[29,30,56]
	HPA/HOPO	HOPO (oxygen-donor) chelators are designed for hard trivalent metal ions; application to Sc^3+^ is chemically plausible but requires isotope-specific validation
	Py-based/H4pypa	Pyridine–picolinate scaffolds provide high thermodynamic stability with ^44^Sc; higher-order derivatives such as H4pypa are structurally related members of this scaffold family; [28]
Mn-52	DOTA/DOTAGA	[77]
	Bispidine/BPPA	[39,41]
	CHX-PYAN	[43]
	TE-series	TE-series data derived from preprint (non-peer-reviewed) study; [41]
	DOTI-Me	[38]
Pb-203	DOTA	[56]
	DOTA-1Py/2Py/3Py	[56,57]
	TCMC (DOTAM)	[56,57,58]
Co-55	NOTA, NODAGA	[56]
	DOTA/DOTAGA	[72,75]
	DiAmSar/DSar	[71]
	TPENYNE	TPENYNE constructs have been evaluated using ^57^Co as a surrogate radionuclide for ^55^Co/^58m^Co, supporting radiochemistry feasibility via click chemistry; chemically plausible for ^55^Co but requires isotope-specific validation; [74]

**Table 4:** Imaging quantification and scanner considerations (limitations differ by isotope: Sc-44, Mn-52, and Co-55 primarily require correction of prompt-γ-induced quantification bias, whereas Tb-149 is limited by reduced sensitivity due to low β^+^ branching).

Isotope	Emission Feature	Quantification Impact	Practical Consideration	Dosimetry Readiness/Translation Stage	References
Tb-149	Low β^+^ yield (~7%)	Low sensitivity due to limited β^+^ yield; minimal prompt-γ-induced quantification bias	Longer acquisitions; sensitivity-aware reconstruction	Preclinical (in vivo therapeutic models)	[12]
Sc-44	β^+^ + 1157 keV prompt γ	Increased randoms; dead-time losses; prompt-γ-induced coincidence contamination	Validate dead-time model; optimize energy window; confirm SUV stability	First-in-human imaging; dosimetry framework emerging	[23,31]
Mn-52	β^+^ (~30%) + multiple γ (744, 936, 1434 keV)	Elevated scatter and random fraction; increased photon burden and potential impact on dose estimates	Count-rate calibration; scatter/random correction verification	Preclinical (antibody and small animal imaging)	[45,77]
Pb-203	γ-emitter (no β^+^)	Not applicable to PET imaging (SPECT-based radionuclide)	Standard SPECT correction workflow (attenuation, scatter, and collimator response)	First-in-human SPECT imaging; clinical theranostic pairing	[48,56]
Co-55	β^+^ + multiple prompt γ (~931, dominant, 1408 keV, additional cascade γ emissions)	Random inflation; dead-time burden; potential SUV bias	Prompt-γ modeling; system calibration at clinical activity levels	Preclinical; limited exploratory human data	[73,76,78]

*Quantitative PET with ^44^Sc, ^52^Mn, and ^55^Co requires validated scatter and random corrections and activity-dependent calibration, as prompt high-energy γ emissions increase random coincidences and dead-time losses at high activity or scanner-dependent count-rate conditions. In contrast, ^149^Tb PET is primarily limited by reduced sensitivity due to its low β^+^ branching (~7%), rather than prompt-γ-induced quantification bias. These factors can influence time–activity curves and absorbed dose estimates, necessitating standardized acquisition and cross-calibration for multicenter dosimetry*.

The second strategy involves coupling production to mass-separation or parent-based collection schemes, whereby **^1^**^49^Tb is isolated indirectly from co-produced radionuclides, most notably its short-lived parent ^149^Dy (t_½_ ≈ 4.2 min). This concept is operationally established at ISOL-class facilities such as CERN-ISOLDE/MEDICIS and is now being coordinated across Europe through the PRISMAP medical-radionuclides network, where high-energy proton-induced spallation followed by on-line mass separation is employed [85]. However, such approaches do not eliminate the underlying accelerator-energy requirement, since ^149^Dy itself is accessed primarily through heavy-ion reactions such as ^142^Nd(^12^C,5n)^149^Dy at ~108–120 MeV, or through GeV-scale (≥1 GeV) proton-induced spallation on tantalum targets [85,86].

^52^Mn and ^55^Co share a common dependence on enriched target material (^52^Cr, ^58^Ni, ^54^Fe), tight control of long-lived impurity channels (^54^Mn, ^57^Co), and the absence of commercial generator systems [35,36,37,66,67,68]. ^203^Pb is limited by the small global number of 24 MeV-class cyclotrons with enriched ^203/205^Tl targetry [51,52,53,54]. ^44g^Sc, while the most infrastructurally accessible of the five, still requires enriched ^44^Ca and careful (p,n) energy-window control to suppress ^44m^Sc co-production [21,22,23]. An overview of current global production capacity, supply reliability, and clinical-readiness status for the five radiometals is presented in Table 5, illustrating the asymmetric translational maturity across the panel.

Taken together, these five radiometals illustrate an emerging spectrum of theranostic strategies in which ^203^Pb and ^44g^Sc are moving closest to routine clinical adoption, ^149^Tb and ^55^Co occupy promising but infrastructure-limited niches, and ^52^Mn provides a distinctive long-half-life PET option for antibody-based vectors. Continued progress in production infrastructure, radiochemistry, and quantitative imaging will determine the pace of their clinical integration [13,53,70,78,79], consistent with the broader principles and ongoing debates in molecular theranostics [87].

## Figures and Tables

**Table 5:** Global production and translational-readiness landscape of the five emerging radiometals (^149^Tb, ^44^Sc, ^52^Mn, ^203^Pb, ^55^Co) (Definitions: Producer denotes facilities performing direct radionuclide production; processing site refers to centers involved in post-irradiation purification and handling; access network/distributor designates coordinating bodies that facilitate isotope availability without primary production. Status indicates the stage of implementation (current, development, or potential), and Grade reflects the level of material supplied (research, preclinical, or clinical potential; see * Footnotes (classification and other global facilities)) [80].

Facility	Role	Status	Grade
Tb-149			
CERN-MEDICIS	Producer	Current	Research
PSI	Processing/collaboration	Current → Future (IMPACT)	Research → Potential
PRISMAP	Access network	Current	Research
TRIUMF	Development	Potential	Research
ARRONAX	Development	Potential	Research
Sc-44 (^44g^Sc) *(via generator, please see footnote “Notable Mentions”)*			
PSI	Producer	Current	Research/Early clinical
UW–Madison	Producer	Current	Research
ARRONAX	Producer	Current	Research
TRIUMF	Producer	Current	Research
PRISMAP	Access network	Current	Research
iThemba LABS	Development	Potential	Research
DOE-NIDC	Distributor	Conditional	Research
Mn-52 (^52g^Mn)			
UW–Madison	Producer	Current	Research
UAB	Producer/Supplier	Current	Research
WashU	Producer	Current	Research
Hevesy-DTU	Producer	Current	Research
PRISMAP	Access network	Current	Research
DOE-NIDC	Distributor	Current	Research
ARRONAX	Development	Potential	Research
INFN-LNL (LARAMED)	Development	Potential	Research
Pb-203			
TRIUMF	Producer/Supplier	Current	Research → Clinical potential
UAB	Producer/Supplier	Current	Research
UAlberta	Producer	Current	Research
DOE-NIDC	Distributor	Current	Research
PRISMAP (ARRONAX, etc.)	Access network	Current	Research/Preclinical
BNL (BLIP)	Development	Potential	Research
BWXT Medical	Commercial development	Pipeline	Clinical potential
Co-55			
UW–Madison	Producer	Current	Research
UAB	Producer/Supplier	Current	Research
DOE-NIDC	Distributor	Current	Research
Odense University Hospital	Producer	Current	Research
Tran Lab- Karolinska Institutet	Development	Potential	Research
ARRONAX	Development	Potential	Research

* Footnotes: Classification of Facilities. Facility classifications are based on publicly available primary literature, institutional reports, and isotope program documentation referenced via the hyperlinks provided for each site. Producer indicates direct radionuclide generation at the listed facility; processing site denotes centers performing post-irradiation separation and formulation; and access network/distributor refers to coordinating entities (e.g., PRISMAP, DOE-NIDC) that facilitate access and distribution without primary production. Status reflects current operational capability versus development or planned capacity at the time of writing and may evolve as new infrastructure becomes available. Grade indicates the typical level of material supplied (research, preclinical, or clinical potential) and does not imply routine GMP-certified commercial availability unless explicitly documented in the cited source. Inclusion in this table reflects demonstrated or reported capability for the specified radionuclide and does not imply continuous supply, regulatory approval, or broad clinical deployment. **Other Potential Global Sites.** The facilities listed in this table represent the principal currently documented sites for production, processing, and distribution of the specified radiometals. In addition to these, a broader network of international infrastructure contributes to isotope development and access. In Europe, this includes PRISMAP-affiliated facilities such as JRC Karlsruhe (Germany), SCK CEN (Belgium), NPI Řež (Czech Republic), and other partner sites, which collectively provide coordinated access, processing, and distribution of non-conventional radionuclides. In Asia, emerging activities at research centers including RIKEN (Japan), the National Institutes for Quantum Science and Technology (QST, Japan), the China Institute of Atomic Energy (CIAE, China), Shanghai Institute of Applied Physics (SINAP, China), and the Korea Atomic Energy Research Institute (KAERI, South Korea) are primarily focused on accelerator-based isotope production research and preclinical development. In Eurasia, accelerator facilities such as the Joint Institute for Nuclear Research (JINR, Dubna) contribute to nuclear-reaction studies and isotope-production R&D relevant to these radionuclides. These additional sites are not individually enumerated in the table, as their roles are predominantly developmental, collaborative, or network-based rather than routine large-scale production or distribution. Accordingly, their status is best classified as development or potential, with material output generally limited to research-grade or preclinical applications. Inclusion of a facility in this table or footnote reflects documented capability or involvement in the radionuclide ecosystem and does not imply continuous supply, regulatory approval, or established clinical-grade production. **(^44g^Sc) Notable Mentions.** ^44^Ti/^44^Sc generator-based production sites—JGU (Germany) and LANL/BNL (via DOE in USA). **Facilities Information—**ARRONAX—Accélérateur pour la Recherche en Radiochimie et Oncologie à Nantes Atlantique (GIP ARRONAX), Saint-Herblain/Nantes, France; BNL—Brookhaven National Laboratory (Medical Isotope Research and Production program, BLIP), Upton, NY, USA; BWXT—BWXT Medical Ltd. (subsidiary of BWX Technologies, Inc.), commercial cGMP cyclotron facilities in Ottawa, ON and at TRIUMF, Vancouver, BC, Canada; CERN-MEDICIS—Medical Isotopes Collected from ISOLDE at CERN (Conseil Européen pour la Recherche Nucléaire/European Organization for Nuclear Research), Meyrin, Geneva, Switzerland; DOE-NIDC—U.S. Department of Energy Isotope Program—National Isotope Development Center, headquartered at Oak Ridge National Laboratory, Oak Ridge, TN, USA (acts as the coordinated supplier/distributor for the DOE-IP University Isotope Network); JGU—Johannes Gutenberg-University Mainz, Institute of Nuclear Chemistry, Mainz, Germany; LANL—Los Alamos National Laboratory, Los Alamos, NM, USA; INFN-LNL—Istituto Nazionale di Fisica Nucleare—Laboratori Nazionali di Legnaro, Legnaro (Padova), Italy. Hosts the LARAMED program (LAboratory of RAdionuclides for MEDicine) on the SPES cyclotron; iThemba—iThemba LABS (Laboratory for Accelerator Based Sciences), National Research Foundation, Cape Town (Faure) and Gauteng, South Africa; LARAMED—LAboratory of RAdionuclides for MEDicine program based at INFN-LNL (Legnaro) and INFN-LNS (Catania), Italy. (Listed separately in the table to flag the medical-isotope subprogram.); Odense University Hospital—Odense Universitets hospital, Department of Nuclear Medicine (Thisgaard group), Odense, Denmark; PRISMAP—PRISMAP—The European Medical Radionuclides Programme, a Horizon-2020/Horizon-Europe consortium of European facilities (CERN-MEDICIS, ILL Grenoble, Arronax, JRC Karlsruhe, PSI, SCK CEN, NPI Řež, ISOLDE-CERN, etc.) that coordinates access to and distribution of novel medical radionuclides; Hevesey-DTU— Hevesy Laboratory in Danmarks Tekniske Universitet, Roskilde, Denmark; PSI—Paul Scherrer Institut, Villigen, Switzerland (Center for Radiopharmaceutical Sciences and the HIPA/Injector II/590 MeV Ring Cyclotron complex; future ISOL production via the IMPACT/TATTOOS project); TRIUMF—TRI-University Meson Facility (now used as a proper name)—Canada’s national particle-accelerator centre, located on the University of British Columbia campus, Vancouver, BC, Canada; UAB—University of Alabama at Birmingham, UAB Cyclotron Facility (Lapi group, GMP-compliant 24 MeV TR-24), Birmingham, AL, USA; UAlberta—University of Alberta, Medical Isotope and Cyclotron Facility (MICF), Edmonton, AB, Canada; UW-Madison—University of Wisconsin–Madison, Department of Medical Physics/Cyclotron Research Group (Engle, Ellison, Nickles legacy), Madison, WI, USA; WashU—Washington University in St. Louis, Mallinckrodt Institute of Radiology Cyclotron Facility, St. Louis, MO, USA; Tran Lab- Karolinska Institutet— TRANslational Theranostics Group, Karolinska Institutet, Sweden.

## Data Availability

No new data were created or analyzed in this study. Data sharing is not applicable.

## Abbreviations

**Abbreviation**	**Full Form**
AAZTA	6-Amino-6-methyl-perhydro-1,4-diazepine-*N*,*N*,*N′*,*N′*-tetraacetic acid
AAZTA5	AAZTA derivative bearing a five-carbon linker for bioconjugation
AG1-X8	Anion-exchange resin (Bio-Rad (Hercules, CA, USA), strong base, 8% cross-linked)
α	Alpha particle/alpha decay
α-PET	Alpha-emitter positron emission tomography (imaging via the β^+^ component of an α-emitter)
α-PRRT	Alpha-emitter peptide-receptor radionuclide therapy
ARRONAX	Accélérateur pour la Recherche en Radiochimie et Oncologie à Nantes-Atlantique
Auger e^−^	Auger electron
β^+^/β^−^	Beta-plus (positron)/beta-minus (electron) decay
BNL	Brookhaven National Laboratory, USA
BPPA	Bispidine-based 6,6′-((6-((bis-pyridin-2-yl-methyl)-amino)pyridine-2,6-diyl)-bis(methylene))-dipicolinic acid
BWXT	BWX Technologies
CD20	Cluster of differentiation 20 (B-cell surface antigen, target of rituximab)
CERN-MEDICIS	European Organization for Nuclear Research—Medical Isotopes Collected from ISOLDE
CIAE	China Institute of Atomic Energy
CT	Computed tomography
DGA	*N*,*N*,*N′*,*N′*-Tetra-n-octyldiglycolamide (extraction-chromatography resin)
DiAmSar (DSar)	Diaminosarcophagine (3,6,10,13,16,19-hexaaza-bicyclo [6.6.6]eicosane-1,8-diamine)
DOE-NIDC	US Department of Energy—National Isotope Development Center
DOTA	1,4,7,10-Tetraazacyclododecane-1,4,7,10-tetraacetic acid
DOTAGA	1,4,7,10-Tetraazacyclododecane-1-glutaric acid-4,7,10-triacetic acid
DOTAM	1,4,7,10-Tetrakis(carbamoylmethyl)-1,4,7,10-tetraazacyclododecane (same compound as TCMC)
DOTANOC	DOTA-1-NaI3-octreotide
DOTATATE	DOTA-(Tyr3)-octreotate
DOTATOC	DOTA-(Tyr3)-octreotide
DOTI-Me	Cyclen-imidazole-based chelator (methylated derivative)
DTPA	Diethylenetriaminepentaacetic acid
EC	Electron capture
EGFR	Epidermal growth factor receptor
Eβ^+^,max	Maximum positron end-point energy
Eγ	Gamma-ray energy
Eα	Alpha-particle energy
EOB	End of bombardment
EU SECURE	European Union Secure Supply of Medical Radioisotopes initiative
FOV	Field of view
γ	Gamma ray/gamma photon
GBq/MBq/kBq	Giga-/Mega-/kilo-becquerel (units of activity)
GMP/cGMP	(Current) Good Manufacturing Practice
GRPR	Gastrin-releasing peptide receptor
HER2	Human epidermal growth factor receptor 2
Hevesey-DTU	Hevesy Laboratory in Danmarks Tekniske Universitet
IAEA	International Atomic Energy Agency
IEDDA	Inverse electron-demand Diels–Alder ligation
IMPACT	Isotope and Muon Production with Advanced Cyclotron and Target technologies (PSI initiative)
INFN-LNL	Istituto Nazionale di Fisica Nucleare—Laboratori Nazionali di Legnaro, Italy
ISOL	Isotope separation on-line
ISOLDE	Isotope Separator On-Line DEvice (CERN)
IT	Isomeric transition
iThemba LABS	iThemba Laboratory for Accelerator-Based Sciences, South Africa
JINR	Joint Institute for Nuclear Research, Dubna, Russia
JRC	Joint Research Centre (European Commission)
KAERI	Korea Atomic Energy Research Institute
LAFOV	Long-axial-field-of-view (total-body PET)
LARAMED	Laboratory of radionuclides for medicine (INFN-LNL)
LET	Linear energy transfer
LM_3_	Somatostatin-receptor antagonist peptide [DOTA-pNO_2_-Phe-c(DCys-Tyr-DAph(Cbm)-Lys-Thr-Cys)-DTyr-NH_2_]
MACROPA	*N*,*N′*-bis[(6-carboxy-2-pyridyl)methyl]-4,13-diaza-18-crown-6
mAb	Monoclonal antibody
mCRPC	Metastatic castration-resistant prostate cancer
MeV/keV	Mega-/kilo-electronvolt
MIP	Maximum intensity projection
MRI	Magnetic resonance imaging
NETs	Neuroendocrine tumors
NODAGA	1,4,7-Triazacyclononane,1-glutaric acid-4,7-diacetic acid
NOTA	1,4,7-Triazacyclononane-1,4,7-triacetic acid
NPI Řež	Nuclear Physics Institute, Řež, Czech Republic
NTSR-1	Neurotensin receptor type 1
P204/P507	Phosphonic/phosphinic-acid liquid–liquid extractants (industrial codes)
PET	Positron emission tomography
PRISMAP	European Medical Radionuclides Programme
PRRT	Peptide-receptor radionuclide therapy
PSC	Pb-specific chelator
PSI	Paul Scherrer Institute, Villigen, Switzerland
PSMA	Prostate-specific membrane antigen
QA/QC	Quality assurance/quality control
QST	National Institutes for Quantum Science and Technology, Japan
RCY	Radiochemical yield
RCP	Radiochemical purity
RIKEN	Rikagaku Kenkyūsho (Institute of Physical and Chemical Research), Japan
ROS	Reactive oxygen species
SCID	Severe combined immunodeficient/immunodeficiency (mouse model)
SCK CEN	Studiecentrum voor Kernenergie—Centre d’Étude de l’énergie Nucléaire, Belgium
SINAP	Shanghai Institute of Applied Physics
SPECT	Single-photon emission computed tomography
SSTR/SSTR2	Somatostatin receptor (subtype 2)
SUV	Standard Uptake Value
TAT	Targeted alpha therapy
TATTOOS	Targeted Alpha Tumor Therapy and Other Oncological Solutions (PSI Programme)
TCMC	1,4,7,10-Tetrakis(carbamoylmethyl)-1,4,7,10-tetraazacyclododecane (also known as DOTAM)
TCO	trans-Cyclooctene
TE-1, TE-5	Pyridinophane-based tetraazacyclododecane derivatives (TE = tetraaza-cyclododecane-ethyl scaffold)
t_½_	Half-life
TLC	Thin-layer chromatography (radio-TLC for radiochemical purity)
TRIUMF	Tri-University Meson Facility, Vancouver, Canada
TPENYNE	*N*,*N*,*N*′,*N*′-tetrakis(2-pyridylmethyl)ethylenediamine (chelator with terminal alkyne handle)
UAB	University of Alabama at Birmingham, USA
UTEVA	Di-pentyl-pentylphosphonate extraction-chromatography resin
UW-Madison	University of Wisconsin—Madison, USA
VIOLET	Clinical trial of [^161^Tb]Tb-PSMA in mCRPC (Phase I/II)
VMT-α-NET	^203^Pb/^212^Pb-labeled SSTR2-targeted peptide for neuroendocrine tumors
WashU	Washington University in St. Louis, USA
XAD-7HP	Amberlite^®^ XAD-7HP polyaromatic adsorbent resin

## References

[1] Watabe T., Hirata K., Iima M., Yanagawa M., Saida T., Sakata A., Ide S., Honda M., Kurokawa R., Nishioka K. (2025). Recent advances in theranostics and oncology PET: Emerging radionuclides and targets. Ann. Nucl. Med..

[2] Tran H.H., Yamaguchi A., Manning H.C. (2025). Radiotheranostic landscape: A review of clinical and preclinical development. Eur. J. Nucl. Med. Mol. Imaging.

[3] Kręcisz P., Stefańska K., Studziński J., Pitucha M., Czylkowska A., Szymański P. (2025). Radiocopper in radiopharmacy and medical use: Current status and perspective. J. Med. Chem..

[4] Meier J.P., Bhuiyan M., Freifelder R., Zhang H.J., Gonzalez L., Pusateri A., Tsai H.M., Leoni L., Ghosh K., Markiewicz E. (2025). Synthesis of DOTA-based ^43^Sc radiopharmaceuticals using cyclotron-produced ^43^Sc as exemplified by [^43^Sc]Sc-PSMA-617 for PSMA PET imaging. Methods Protoc..

[5] Mapanao A.K., Busslinger S.D., Mehta A., Kegler K., Favaretto C., Grundler P.V., Talip Z., Köster U., Johnston K., Schibli R. (2025). Preclinical investigation of [^149^Tb]Tb-DOTATATE and [^149^Tb]Tb-DOTA-LM_3_ for tumor-targeted alpha therapy. Eur. J. Nucl. Med. Mol. Imaging.

[6] Abdlkadir A.S., Rosar F., Jalilian A., Moghrabi S., Al-Balooshi B., Rabei O., Kairemo K., Al-Ibraheem A. (2025). Harnessing terbium radioisotopes for clinical advancements: A systematic review. Nucl. Med. Mol. Imaging.

[7] Kuyumcu S., Şanlı Y. (2025). Reflections on Terbium-149: Advancing preclinical research in targeted alpha therapy. Eur. J. Nucl. Med. Mol. Imaging.

[8] Bills L.A., McIntosh A.B., Morrell J.T., Adsley P., Abbott A.D., Rojas D.C., Garcia-Duarte J., Gott M.D., Hagel K., Hankins T. (2025). Cross sections of ^147–149^Sm(^6^Li,x) reactions for the production of ^149^Tb for targeted alpha therapy. Sci. Rep..

[9] Roustaei H., D’Alessandria C., Decristoforo C. (2025). Navigating the safety profile of Actinium-225 targeted alpha therapy: A comprehensive review. Clin. Transl. Imaging.

[10] Ma F., Zhang W., Xu Z., Yang D., Wang G. (2025). Separation and purification of gadolinium, terbium and dysprosium by P204-P507 solvent impregnated resin in nitric acid system. J. Mol. Liq..

[11] Di Iorio V., Sarnelli A., Boschi S., Sansovini M., Genovese R.M., Stefanescu C., Ghizdovat V., Jalloul W., Young J., Sosabowski J. (2025). Recommendations on the clinical application and future potential of α-particle therapy: A comprehensive review of the results from the SECURE Project. Pharmaceuticals.

[12] Müller C., Vermeulen C., Köster U., Johnston K., Türler A., Schibli R., van der Meulen N.P. (2017). Alpha-PET with terbium-149: Evidence and perspectives for radiotheranostics. EJNMMI Radiopharm. Chem..

[13] Umbricht C.A., Köster U., Bernhardt P., Gracheva N., Johnston K., Schibli R., van der Meulen N.P., Müller C. (2019). Alpha-PET for Prostate Cancer: Preclinical investigation using ^149^Tb-PSMA-617. Sci. Rep..

[14] Lacerda S., de Kruijff R.M., Djanashvili K. (2025). The advancement of targeted alpha therapy and the role of click chemistry therein. Molecules.

[15] D’Onofrio A., Silva F., Gano L., Karczmarczyk U., Mikołajczak R., Garnuszek P., Paulo A. (2021). Clickable radiocomplexes with trivalent radiometals for cancer theranostics: In vitro and in vivo studies. Front. Med..

[16] Adhikari K., Vanermen M., Da Silva G., Van den Wyngaert T., Augustyns K., Elvas F. (2024). Trans-cyclooctene-a Swiss army knife for bioorthogonal chemistry: Exploring the synthesis, reactivity, and applications in biomedical breakthroughs. EJNMMI Radiopharm. Chem..

[17] Ji Y., Wang M., Yao Z., Ma J., Zhang C. (2026). The potential and research progress of ^161^Tb-based targeted radionuclide therapy. Clin. Transl. Imaging.

[18] Beyer G.J., Miederer M., Vranjes-Durić S., Comor J.J., Künzi G., Hartley O., Senekowitsch-Schmidtke R., Soloviev D., Buchegger F. (2004). Targeted alpha therapy in vivo: Direct evidence for single cancer cell kill using ^149^Tb-rituximab. Eur. J. Nucl. Med. Mol. Imaging.

[19] Buteau J.P., Kostos L., Jackson P.A., Xie J., Haskali M.B., Alipour R., McIntosh L.E., Emmerson B., MacFarlane L., Martin C.A. (2025). First-in-human results of terbium-161 [^161^Tb]Tb-PSMA-I&T dual β/Auger radioligand therapy in patients with metastatic castration-resistant prostate cancer (VIOLET): A single-centre, single-arm, phase 1/2 study. Lancet Oncol..

[20] Chirindel A., Nicolas G.P., Westerbergh F., McDougall L., Schmid D.E., Geistlich S., Tschan V.J., Busslinger S.D., Fokkema A., Aceto N. (2025). First-in-human administration of [^161^Tb]Tb-SibuDAB and comparative dosimetry with standard [^177^Lu]Lu-PSMA-I&T as part of the PROGNOSTICS phase Ia study. Eur. J. Nucl. Med. Mol. Imaging.

[21] Becker K.V., Aluicio-Sarduy E., Bradshaw T., Hurley S.A., Olson A.P., Barrett K.E., Batterton J., Ellison P.A., Barnhart T.E., Pirasteh A. (2023). Cyclotron production of ^43^Sc and ^44g^Sc from enriched ^42^CaO, ^43^CaO, and ^44^CaO targets. Front. Chem..

[22] Schmidt C.E., Groveman S., Sanders V.A., Cutler C.S., Shusterman J.A., Deri M.A. (2024). Development of a SnO_2_-based ^44^Ti/^44^Sc generator for medical applications. J. Chromatogr. A.

[23] van der Meulen N.P., Hasler R., Talip Z., Grundler P.V., Favaretto C., Umbricht C.A., Müller C., Dellepiane G., Carzaniga T.S., Braccini S. (2020). Developments toward the Implementation of ^44^Sc Production at a Medical Cyclotron. Molecules.

[24] Schmidt C.E., Gajecki L., Deri M.A., Sanders V.A. (2023). Current state of ^44^Ti/^44^Sc radionuclide generator systems and separation chemistry. Curr. Radiopharm..

[25] Chai X., Nawar M.F., Lüthi M., Zingg R., Kottler C., Türler A. (2025). Towards a dry separation method of radioscandium from bulk amounts of titanium. J. Radioanal. Nucl. Chem..

[26] International Atomic Energy Agency (IAEA) (2024). Photonuclear Production of Radioisotopes; IAEA-TECDOC-2051.

[27] Whetter J.N., Śmiłowicz D., Becker K.V., Aluicio-Sarduy E., Kelderman C.A.A., Koller A.J., Glaser O.M., Marlin A., Ahn S.H., Kretowicz M.N. (2024). Phosphonate-based aza-macrocycle ligands for low-temperature, stable chelation of medicinally relevant rare earth radiometals and radiofluorination. J. Am. Chem. Soc..

[28] Li L., Jaraquemada-Peláez M.G., Aluicio-Sarduy E., Wang X., Jiang D., Sakheie M., Kuo H.T., Barnhart T.E., Cai W., Radchenko V. (2020). [^nat/44^Sc(pypa)]: Thermodynamic stability, radiolabeling, and biodistribution of a prostate-specific-membrane-antigen-targeting conjugate. Inorg. Chem..

[29] Sinnes J.P., Bauder-Wüst U., Schäfer M., Moon E.S., Kopka K., Rösch F. (2020). ^68^Ga, ^44^Sc and ^177^Lu-labeled AAZTA5-PSMA-617: Synthesis, radiolabeling, stability and cell binding compared to DOTA-PSMA-617 analogues. EJNMMI Radiopharm. Chem..

[30] Bera A., Glaser O.M., Aluicio-Sarduy E., Engle J.W., Meimetis L.G., Boros E., Śmiłowicz D. (2025). Adapting solid phase radiometalation photorelease to the synthesis of ^44^Sc and ^177^Lu radiopharmaceuticals. Mol. Pharm..

[31] Singh A., van der Meulen N.P., Müller C., Klette I., Kulkarni H.R., Türler A., Schibli R., Baum R.P. (2017). First-in-human PET/CT imaging of metastatic neuroendocrine neoplasms with cyclotron-produced ^44^Sc-DOTATOC: A proof-of-concept study. Cancer Biother. Radiopharm..

[32] Eppard E., de la Fuente A., Benešová M., Khawar A., Bundschuh R.A., Gärtner F.C., Kreppel B., Kopka K., Essler M., Rösch F. (2017). Clinical translation and first in-human use of [^44^Sc]Sc-PSMA-617 for PET imaging of metastasized castrate-resistant prostate cancer. Theranostics.

[33] Khawar A., Eppard E., Sinnes J.P., Roesch F., Ahmadzadehfar H., Kürpig S., Meisenheimer M., Gaertner F.C., Essler M., Bundschuh R.A. (2018). [^44^Sc]Sc-PSMA-617 biodistribution and dosimetry in patients with metastatic castration-resistant prostate carcinoma. Clin. Nucl. Med..

[34] Bailey D.L., Willowson K.P., O’Keefe G., Goodman S., Patford S., McGill G., Pattison D.A., Scott A.M., I-FIRST Investigators and ARTnet (2025). A method for validating PET and SPECT cameras for quantitative clinical imaging trials using novel radionuclides. J. Nucl. Med..

[35] Porto F., Cisternino S., Cazzola E., Speltri G., Mou L., Boschi A., Marvelli L., Di Domenico G., Pagnoni A., De Dominicis L. (2024). Cyclotron production of manganese-52: A promising avenue for multimodal PET/MRI imaging. EJNMMI Radiopharm. Chem..

[36] Pyles J.M., Omweri J.M., Lapi S.E. (2023). Natural and enriched Cr target development for production of Manganese-52. Sci. Rep..

[37] Kretowicz M.N., Barrett K.E., Barnhart T.E., Engle J.W. (2023). Recycling of ^52^Cr electroplated targets for ^52g^Mn production. Appl. Radiat. Isot..

[38] Hierlmeier I., Marino N., Schreck M.V., Schneider L., Maus S., Barrett K., Kretowicz M., Engle J.W., Pierri G., Ezziddin S. (2024). Radiochemistry and complex formation of the cyclen-derived chelator DOTI-Me with Mn^2+^, Cu^2+^, Zn^2+^, Ga^3+^, In^3+^, Tb^3+^, and Lu^3+^. Inorg. Chem..

[39] Toàn N.M., Vágner A., Nagy G., Ország G., Nagy T., Csikos C., Váradi B., Sajtos G.Z., Kapus I., Szoboszlai Z. (2024). [^52^Mn]Mn-BPPA-Trastuzumab: A promising HER2-specific PET radiotracer. J. Med. Chem..

[40] Omweri J.M., Houson H.A., Lynch S.E., Tekin V., Sorace A.G., Lapi S.E. (2025). PET imaging of [^52^Mn]Mn-DOTATATE and [^52^Mn]Mn-DOTA-JR11. Sci. Rep..

[41] El Sayed T., Terpstra K., Whetter J., Xu K., Zhu L., Chakrabarti S., Marlin A., Wessel A., Majumdar S., Sutton B. (2026). Pyridinophane ligands: An attractive chelator platform for Mn-Based imaging agents. J. Med. Chem..

[42] Omweri J.M., Saini S., Houson H.A., Tekin V., Pyles J.M., Parker C.C., Lapi S.E. (2024). Development of ^52^Mn labeled trastuzumab for extended time point pet imaging of HER2. Mol. Imaging Biol..

[43] Harriswangler C., Omweri J.M., Saini S., Valencia L., Esteban-Gómez D., Ranga M., Guidolin N., Baranyai Z., Lapi S.E., Platas-Iglesias C. (2024). Improving the in vivo stability of [^52^Mn]Mn(II) complexes with 18-membered macrocyclic chelators for PET imaging. J. Med. Chem..

[44] Ma X., He C., Wang Y., Cao X., Jin Z., Ge Y., Cao Z., An M., Hao L. (2025). Mechanisms and applications of manganese-based nanomaterials in tumor diagnosis and therapy. Biomater. Res..

[45] Graves S.A., Hernandez R., Fonslet J., England C.G., Valdovinos H.F., Ellison P.A., Barnhart T.E., Elema D.R., Theuer C.P., Cai W. (2015). Novel preparation methods of ^52^Mn for immuno-PET imaging. Bioconjug. Chem..

[46] Hernandez R., Graves S.A., Gregg T., VanDeusen H.R., Fenske R.J., Wienkes H.N., England C.G., Valdovinos H.F., Jeffery J.J., Barnhart T.E. (2017). Radiomanganese PET detects changes in functional β-cell mass in mouse models of diabetes. Diabetes.

[47] Napieczynska H., Severin G.W., Fonslet J., Wiehr S., Menegakis A., Pichler B.J., Calaminus C. (2017). Imaging neuronal pathways with ^52^Mn PET: Toxicity evaluation in rats. Neuroimage.

[48] Kästner D., Hartmann H., Freudenberg R., Pretze M., Brogsitter C., Schultz M.K., Kotzerke J., Michler E. (2025). Gamma camera imaging characteristics of ^203/212^Pb as a theranostic pair for targeted alpha therapy: A feasibility study. EJNMMI Phys..

[49] Müller D., Herrmann H., Schultz M.K., Solbach C., Ettrich T., Prasad V. (2023). ^203^Pb-VMT-α-NET scintigraphy of a patient with neuroendocrine tumor. Clin. Nucl. Med..

[50] Lee D., Li M., Liu D., Baumhover N.J., Sagastume E.A., Marks B.M., Rastogi P., Pigge F.C., Menda Y., Johnson F.L. (2024). Structural modifications toward improved lead-203/lead-212 peptide-based image-guided alpha-particle radiopharmaceutical therapies for neuroendocrine tumors. Eur. J. Nucl. Med. Mol. Imaging.

[51] Saini S., Bartels J.L., Appiah J.K., Rider J.H., Baumhover N., Schultz M.K., Lapi S.E. (2023). Optimized methods for the production of high-purity ^203^Pb using electroplated thallium targets. J. Nucl. Med..

[52] Jurisson S.S., Heather H., Li Y., Scott D.W. (2024). Production of High Specific Activity ^72^Se/^72^As, ^117m^Sn and ^203^Pb for Research and Clinical Applications: Effective Target Design, Recycling of Target Material and Radioisotope Separation.

[53] Rosales J.J., Domínguez M.L., Sancho L., Prieto E., de Arcocha M., Torres I., Roteta A., Ramos R., Quincoces G. (2025). State of the art and future perspectives of new radionuclides in Nuclear Medicine. Rev. Esp. Med. Nucl. Imagen Mol..

[54] Blanco J.R., Edwards T.S., Irvin T.E., Kleinfeldt C.R., Lapi S.E., Severin G.W. (2025). Cyclotron production of a ^204^Bi/^204m^Pb generator by 24 MeV proton irradiation of natural Pb foil targets. Appl. Radiat. Isot..

[55] Ramonaheng K., Qebetu M., Banda K., Goorhoo P., Legodi K., Mdanda S., Sibiya S., Mzizi Y., Ndlovu H., Kabunda J. (2025). Advances in dosimetry and imaging for ^203^Pb and ^212^Pb. Semin. Nucl. Med..

[56] Krol V.E., Pandey M.K., Srivastava V., Vatsa R. (2025). Chelator Design and Radiolabeling Chemistry of Radiometals. Targeted Radiopharmaceuticals and Imaging: Development Challenges and Opportunities.

[57] McNeil B.L., Robertson A.K.H., Fu W., Yang H., Hoehr C., Ramogida C.F., Schaffer P. (2021). Production, purification, and radiolabeling of the ^203^Pb/^212^Pb theranostic pair. EJNMMI Radiopharm. Chem..

[58] Vaughn B.A., Veach D.R., Vargas D.B., Seo S., Punzalan B., Rinne S.S., Fung E.K., Xu H., Guo H.-F., Yang G. (2026). Preclinical ^203/212^Pb-DOTA based pre-targeted radioimmunotherapy in nude mice bearing established human colorectal cancer xenografts. J. Nucl. Med..

[59] Sarrami N., Nelson B., Leier S., Wilson J., Chan C., Meens J., Komal T., Ailles L., Wuest M., Schultz M. (2024). SPECT/CT imaging of EGFR-positive head and neck squamous cell carcinoma patient-derived xenografts with ^203^Pb-PSC-panitumumab in NRG mice. EJNMMI Radiopharm. Chem..

[60] Gao Y., Ivanovich K., Medin S., Pian B., MacMillan S.N., Schmitz A.M., Wilson J.J. (2025). 18-membered macrocycle appended on resin for selective rare earth element extraction and separation. Commun. Chem..

[61] Thakral P., Sen I.B., Das S.S., Schultz M.K., Kumari J., Virupakshappa C.B., Malik D. (2024). Lead-203 VMT-α-neuroendocrine tumor scintigraphy: A promising theranostics agent. Indian. J. Nucl. Med..

[62] Dos Santos J.C., Schäfer M., Bauder-Wüst U., Lehnert W., Leotta K., Morgenstern A., Kopka K., Haberkorn U., Mier W., Kratochwil C. (2019). Development and dosimetry of ^203^Pb/^212^Pb-labelled PSMA ligands: Bringing “the lead” into PSMA-targeted alpha therapy?. Eur. J. Nucl. Med. Mol. Imaging.

[63] Michler E., Kästner D., Pretze M., Hartmann H., Freudenberg R., Schultz M.K., Bundschuh R.A., Kotzerke J., Brogsitter C. (2025). [^203/212^Pb]Pb-VMT-α-NET as a novel theranostic agent for targeted alpha radiotherapy–first clinical experience. Eur. J. Nucl. Med. Mol. Imaging.

[64] Graves S.A., Bushnell D.L., Schultz M.K., Jain S., Bodeker K.L., Menda Y. (2026). A phase 0 imaging trial of [^203^Pb]Pb-VMT-α-NET to enable dosimetry and treatment planning for refractory or relapsed metastatic neuroendocrine tumors with [^212^Pb]Pb-VMT-α-NET. J. Nucl. Med..

[65] Leupe H., Cauwenbergh M., Cleeren F., Dekervel J., Verslype C., Deroose C.M. (2025). Clinical experience with targeted alpha-emitter peptide-receptor radionuclide therapy (α-PRRT) for somatostatin-receptor-positive neuroendocrine tumors. Pharmaceuticals.

[66] Boros E., Packard A.B. (2019). Radioactive Transition Metals for Imaging and Therapy. Chem. Rev..

[67] Valdovinos H.F., Hernandez R., Graves S., Ellison P.A., Barnhart T.E., Theuer C.P., Engle J.W., Cai W., Nickles R.J. (2017). Cyclotron production and radiochemical separation of ^55^Co and ^58m^Co from ^54^Fe, ^58^Ni and ^57^Fe targets. Appl. Radiat. Isot..

[68] Barrett K.E., Houson H.A., Lin W., Lapi S.E., Engle J.W. (2021). Production, purification, and applications of a potential theranostic pair: Cobalt-55 and Cobalt-58m. Diagnostics.

[69] Sanwick A.M., Chaple I.F. (2025). Radiocobalt theranostic applications: Current landscape, challenges, and future directions. Front. Nucl. Med..

[70] Siikanen J., Milton S., Bratteby K., Lin W., Engle J.W., Jussing E., Tran T.A. (2025). Rapid fabrication and dissolution of pressed ^58^Ni/Mg matrix targets for ^55^Co production. EJNMMI Radiopharm. Chem..

[71] Lin W., Fonseca Cabrera G.O., Aluicio-Sarduy E., Barnhart T.E., Mixdorf J.C., Li Z., Wu Z., Engle J.W. (2024). Radiolabeling diaminosarcophagine with cyclotron-produced cobalt-55 and [^55^Co]Co-NT-sarcage as a proof of concept in a murine xenograft model. Bioconjug Chem..

[72] Mitran B., Thisgaard H., Rinne S., Dam J.H., Azami F., Tolmachev V., Orlova A., Rosenström U. (2019). Selection of an optimal macrocyclic chelator improves the imaging of prostate cancer using cobalt-labeled GRPR antagonist RM26. Sci. Rep..

[73] Cabrera G.O.F., Ma X., Lin W., Zhang T., Zhao W., Pan L., Li X., Barnhart T.E., Aluicio-Sarduy E., Deng H. (2024). Synthesis of ^64^Cu-, ^55^Co-, and ^68^Ga-labeled radiopharmaceuticals targeting neurotensin receptor-1 for theranostics: Adjusting in vivo distribution using multiamine macrocycles. J. Nucl. Med..

[74] Gé L.G., Danielsen M.B., Nielsen A.Y., Skavenborg M.L., Langkjær N., Thisgaard H., McKenzie C.J. (2025). Radiocobalt-labeling of a polypyridylamine chelate conjugated to GE11 for EGFR-targeted theranostics. Molecules.

[75] Baun C., Dam J.H., Hildebrandt M.G., Ewald J.D., Kristensen B.W., Gammelsrød V.S., Olsen B.B., Thisgaard H. (2023). Preclinical evaluation of [^58m^Co]Co-DOTA-PSMA-617 for auger electron therapy of prostate cancer. Sci. Rep..

[76] Stevens H., Jansen H.M., De Reuck J., Lemmerling M., Strijckmans K., Goethals P., Lemahieu I., de Jong B.M., Willemsen A.T., Korf J. (1999). Cobalt-55 (^55^Co) as a PET-tracer in stroke, compared with blood flow, oxygen metabolism, blood volume and gadolinium-MRI. J. Neurol. Sci..

[77] Price E.W., Orvig C. (2014). Matching chelators to radiometals for radiopharmaceuticals. Chem. Soc. Rev..

[78] Andersen T.L., Baun C., Olsen B.B., Dam J.H., Thisgaard H. (2020). Improving Contrast and Detectability: Imaging with [^55^Co]Co-DOTATATE in Comparison with [^64^Cu]Cu-DOTATATE and [^68^Ga]Ga-DOTATATE. J. Nucl. Med..

[79] van der Meulen N.P., Bunka M., Domnanich K.A., Müller C., Haller S., Vermeulen C., Türler A., Schibli R. (2015). Cyclotron production of ^44^Sc: From bench to bedside. Nucl. Med. Biol..

[80] Link for Sites (Current and Potential Producers, Suppliers, Distributors): Sites_information_binder_Tb-149_Sc-44_Pb-203_Mn-52_Co-55. https://acrobat.adobe.com/id/urn:aaid:sc:US:76879b0b-886c-448f-ae54-ea3d57dce170.

[81] Nawar M.F., Selim A.A., Essa B.M., El-Daoushy A.F., Swidan M.M., Chambers C.G., Al Qahtani M.H., Smith C.J., Sakr T.M. (2025). Actinium-225/Bismuth-213 as potential leaders for targeted alpha therapy: Current supply, application barriers, and future prospects. Cancers.

[82] Steyn G.F., Vermeulen C., Szelecsényi F., Kovács Z., Hohn A., van der Meulen N.P., Schibli R., van der Walt T.N. (2014). Cross sections of proton-induced reactions on ^152^Gd, ^155^Gd and ^159^Tb with emphasis on the production of selected Tb radionuclides. Nucl. Instrum. Methods Phys. Res. B.

[83] Favaretto C., Grundler P.V., Talip Z., Köster U., Johnston K., Busslinger S.D., Sprung P., Hillhouse C.C., Eichler R., Schibli R. (2024). Terbium-149 production: A focus on yield and quality improvement towards preclinical application. Sci. Rep..

[84] Moiseeva A.N., Aliev R.A., Unezhev V.N., Zagryadskiy V.A., Latushkin S.T., Aksenov N.V., Gustova N.S., Voronuk M.G., Starodub G.Y., Ogloblin A.A. (2020). Cross section measurements of ^151^Eu(^3^He,5n) reaction: New opportunities for medical alpha emitter ^149^Tb production. Sci. Rep..

[85] Moiseeva A.N., Favaretto C., Talip Z., Grundler P.V., van der Meulen N.P. (2024). Terbium sisters: Current development status and upscaling opportunities. Front. Nucl. Med..

[86] Beyer G.J., Čomor J.J., Daković M., Soloviev D., Tamburella C., Hagebø E., Allan B., Dmitriev S.N., Zaitseva N.G., ISOLDE Collaboration (2002). Production routes of the alpha emitting ^149^Tb for medical application. Radiochim. Acta.

[87] Currie G.M. (2025). Molecular theranostics: Principles, challenges and controversies. J. Med. Radiat. Sci..

